# Ribosome-associated chaperone DNAJC2 titrates translation flux to alleviate the burden of co-translational quality control

**DOI:** 10.1016/j.mocell.2026.100407

**Published:** 2026-10-14

**Authors:** Jongmin Park, Youngim Yu, Heeju Park, Hyunwoo Bang, Jongbin Lee, Chunghun Lim

**Affiliations:** 1 Department of Biological Sciences, Korea Advanced Institute of Science and Technology, Daejeon 34141, Republic of Korea; 2 Department of Biological Sciences, Ulsan National Institute of Science and Technology, Ulsan 44919, Republic of Korea; 3 KAIST Stem Cell Center, Korea Advanced Institute of Science and Technology, Daejeon 34141, Republic of Korea; 4 Graduate School of Stem Cell and Regenerative Biology, Korea Advanced Institute of Science and Technology, Daejeon 34141, Republic of Korea

**Keywords:** DNAJC2, Ribosome-associated complex, Ribosome-associated quality control, Ribotoxic stress response, Translational flux

## Abstract

Ribosome collisions during aberrant translation activate ribosome-associated quality control (RQC) to resolve stalled ribosome complexes and triage incomplete nascent chains. How this co-translational surveillance is coordinated with other ribosome-associated activities remains unclear. Here, we identify the ribosome-associated Hsp40 chaperone DNAJC2 that restrains translational flux and limits RQC engagement during collision-prone translation. Genetic epistasis analysis revealed that DNAJC2 titrates the flux of aberrant translation intermediates through the canonical RQC pathway, from the collision-sensing RQC factor ZNF598 to the downstream NEMF-LTN1 E3 ligase complex for proteasomal degradation. DNAJC2 depletion further sensitized cells to collision-induced ribotoxic stress response (RSR), indicating increased vulnerability to global translation stress. Mechanistically, loss of DNAJC2 accelerated translation elongation, thereby promoting ribosome collisions and ZNF598-dependent ribosome disassembly. Accordingly, reducing translational flux with the translation inhibitor homoharringtonine or a huntingtin upstream ORF rescued DNAJC2-depletion phenotypes during RQC-sensitive poly(A)-mediated translational stalling. Together, we propose that DNAJC2 acts as a translational rheostat to buffer ribosome collisions, prevent RQC overload, and set the threshold for RSR activation.

## Graphical Abstract

**Figure:**
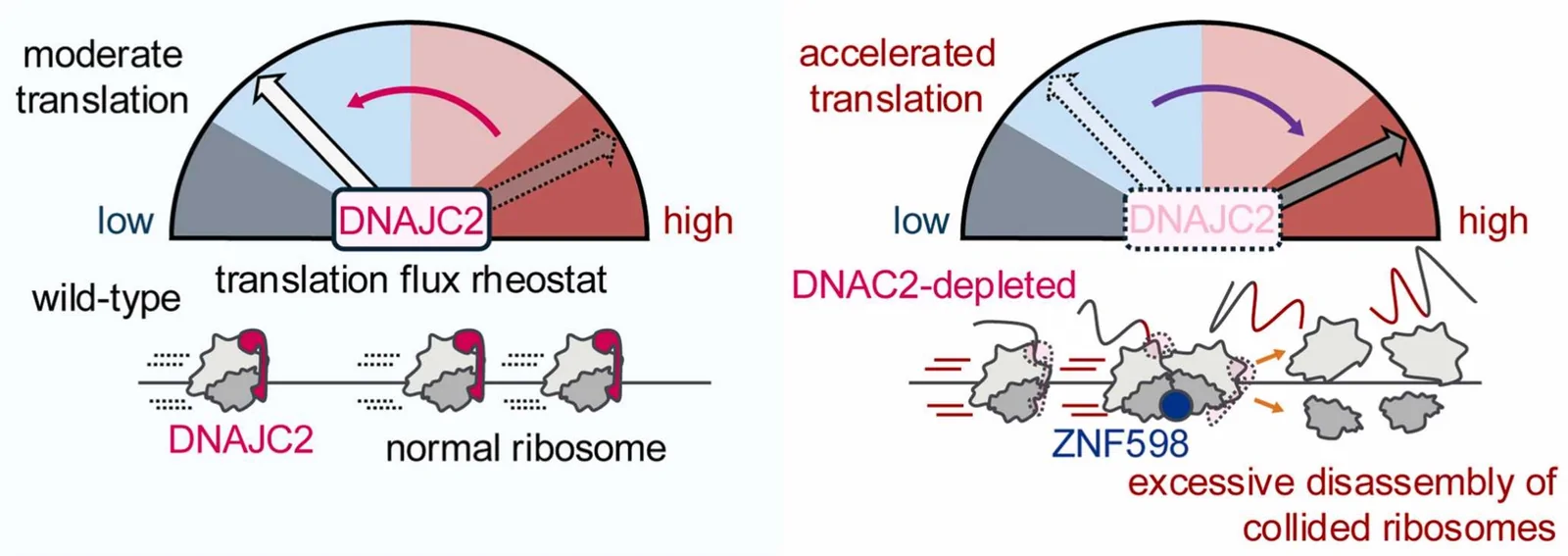

## BACKGROUND

Gene expression decodes genetic information to generate and amplify molecular effectors with diverse biological activities. Quality control of the gene products operates at nearly every stage of the decoding process to sustain cellular physiology (Chen et al., 2011a, D'Orazio and Green, 2021, Doma and Parker, 2007, Isken and Maquat, 2007, Lindahl and Wood, 1999, Maquat and Carmichael, 2001, Schmid and Jensen, 2010, Wolff et al., 2014, Yan and Zaher, 2019). In particular, the ribosome-associated quality control (RQC) pathway senses ribosome collisions as a sign of atypical mRNA translation, resolving aberrant translation intermediates for proteostasis while rescuing stalled ribosomes for recycling (Brandman and Hegde, 2016, Brandman et al., 2012, Buskirk and Green, 2017, Filbeck et al., 2022, Ford et al., 2024, Garshott et al., 2021, Guydosh and Green, 2014, Inada and Aiba, 2005, Joazeiro, 2019, Muller et al., 2025, Park et al., 2021a, Simms et al., 2017, Vind et al., 2020a). Consistent with the significance of co-translational quality control, growing evidence indicates that ribosome collisions and RQC activity are involved in various physiological contexts, including development, disease, and aging (Albanese et al., 2006, Chu et al., 2009, Craig et al., 2003, Endo et al., 2023, Kramer et al., 2019, Lee et al., 2025, Li et al., 2020, Martin et al., 2020, Park et al., 2021b, Park et al., 2026, Pechmann et al., 2013, Rimal et al., 2021, Robinson et al., 2022, Rodschinka et al., 2025, Stein et al., 2022, Terrey et al., 2020, Turner and Varshavsky, 2000, Udagawa et al., 2021, Vind et al., 2020, Vind et al., 2024, Zaher and Mosammaparast, 2025).

Ribosome stalling occurs when ribosomes face a distinct translational environment, potentially leading to a collisional event between 2 consecutive ribosomes on the same mRNA molecule. Collision-inducing conditions include aberrant mRNA templates (eg, poly-A tracts encoding polylysine residues; improperly processed or damaged mRNAs), atypical translation episodes (eg, translation of stalling-inducing nascent polypeptides; stop-codon readthrough), and cellular stress (eg, amino acid deprivation). During RQC, the rate-limiting E3 ligase ZNF598 recognizes collided ribosomes and ubiquitinates 40S ribosomal proteins (eg, RPS10, RPS20) (Garzia et al., 2017, Juszkiewicz and Chandrasekaran, 2018, Juszkiewicz and Hegde, 2017, Sundaramoorthy and Leonard, 2017). The ubiquitinated 40S subunit subsequently recruits the RQC-triggering (RQT) complex (ie, ASCC2-ASCC3-TRIP4 complex) to dissociate the stalled 80S ribosome into the large (60S) and small (40S) subunits (Hashimoto et al., 2020). NEMF then binds the 60S-nascent chain complex to prevent its reassociation with the 40S subunit (Kostova et al., 2017, Lyumkis et al., 2014, Shao et al., 2015, Shen et al., 2015, Sitron and Brandman, 2019) while mediating the addition of nontemplated alanine-threonine tails to the C-terminus of the nascent chain entrapped in the 60S (Choe et al., 2016, Izawa and Park, 2017, Kostova et al., 2017, Shen et al., 2015, Tesina and Ebine, 2023, Udagawa et al., 2021, Yonashiro et al., 2016). NEMF also supports stable binding of another E3 ligase LTN1 to the 60S-nascent chain complex, facilitating ubiquitination of the RQC substrate (ie, aberrantly terminated nascent chain) for proteasomal decay (Bengtson and Joazeiro, 2010, Brandman et al., 2012, Shao et al., 2015).

Ribosome-associated chaperones belong to another class of co-translational regulators that sustain proper folding of nascent chains at the ribosome tunnel exit during translation elongation (Deuerling et al., 2019, Gamerdinger and Deuerling, 2024, Gautschi et al., 2001, Kramer et al., 2019, Morales-Polanco et al., 2022). Two types of ribosomal chaperone complexes have been discovered in eukaryotes: the nascent polypeptide-associated complex (NAC) and the ribosome-associated complex (RAC), each consisting of 2 heterodimeric components (ie, NACA and BTF3/NACB for NAC; DNAJC2/ZUO1/MPP11/ZRF1 and HSPA14/HSP70L1 for RAC) (Deuerling et al., 2019). These ribosome-associated chaperone complexes play complementary yet distinct roles in the co-translational folding of diverse nascent chains while both executing additional functions during translation. For example, intracellular transport of the nuclear-encoded organellar proteins can occur co-translationally, and NAC helps ensure their accurate targeting to the ER or mitochondria during translation (Gamerdinger et al., 2015, Hotokezaka et al., 2009, Jomaa et al., 2022). NAC also recruits methionine aminopeptidases to the ribosomal tunnel exit for N-terminal methionine excision from nascent polypeptides (Gamerdinger et al., 2023). On the other hand, yeast genetic studies have revealed that RAC suppresses premature termination at ribosome-stalling sequences and stop-codon readthrough while supporting programmed ribosomal frameshifting (Chiabudini et al., 2012, Chiabudini et al., 2014, Gribling-Burrer et al., 2019, Jaiswal et al., 2011, Lee et al., 2016, Muldoon-Jacobs and Dinman, 2006, Rakwalska and Rospert, 2004, Zhang et al., 2017). Structural and genetic studies further suggest that yeast RAC likely exerts these effects on translation fidelity through its binding across the ribosome subunits. Cryo-electron microscopy revealed the elongated structure of yeast RAC that spans both the 60S and 40S ribosomal subunits. In particular, Zuo1 (a yeast homolog of DNAJC2) interacts with an rRNA expansion segment at the 40S subunit that is structurally linked to the catalytic center of the ribosome (Chen et al., 2022, Kisonaite et al., 2023, Lee et al., 2016, Zhang et al., 2014). This mode of ribosomal association likely enables RAC to restrain ribosomal translocation while coupling translation elongation to co-translational folding of nascent chains (Kisonaite et al., 2023, Zhang et al., 2014).

Despite these advances, ribosomal RAC functions remain much less clearly understood in mammals. RAC homologs display distinct domain structures between yeast and mammals, and the yeast RAC operates together with the ribosome-bound Hsp70 Ssb (Ssb1/2) to support co-translational chaperone function (Deuerling et al., 2019, Kramer et al., 2019). It therefore remains unclear whether mammalian RAC similarly coordinates distinct ribosomal activities at the nascent chain exit tunnel, including translation elongation, nascent chain handling, and co-translational surveillance in higher eukaryotes. This question is particularly relevant to RQC since RQC must be selectively engaged at problematic ribosome stalls while avoiding excessive activation during normally slowed translation events. Defining how ribosome-associated chaperones are functionally coordinated with the RQC machinery is therefore essential for understanding how cells balance productive protein synthesis with co-translational quality control. Here, we demonstrate functional crosstalk between RAC and the RQC pathway, identifying the human RAC component DNAJC2 as an upstream regulator that tunes RQC engagement during collision-prone translation.

## MATERIALS AND METHODS

### Cell Culture, Transfection, and Stable Cell Line Generation

Human neuroblastoma SH-SY5Y cells (ATCC, CRL-2266) were cultured in Dulbecco’s Modified Eagle Medium (HyClone and Welgene) supplemented with 10% fetal bovine serum (Gibco), 100 U/ml penicillin, and 100 µg/ml streptomycin (Gibco and Hyclone). Cell cultures were maintained at 37°C in a humidified incubator with 95% air and 5% CO_2_. Plasmid DNAs and siRNAs were transiently transfected into cultured cells using polyethylenimine (Polyscience) or Lipofectamine RNAiMAX transfection reagent (Thermo Fisher Scientific) according to the manufacturers’ instructions. Unless otherwise indicated, SH-SY5Y cells were harvested 48 hours after DNA transfection or 72 hours after siRNA transfection for subsequent analyses. Genomic deletions of *ZNF598*, *NEMF*, and *LTN1* loci in SH-SY5Y cells (*ZNF598*^KO^*, LTN1*^KO^, and *NEMF*^KO^ cell lines) have been described previously (Park et al., 2021b). To generate JNK-KTR stable lines or *ZAK*^KO^ cell lines, lentiviral vectors were co-transfected with 3rd-generation packaging plasmids (pLP1, pLP2, and pVSV-G) into human embryonic kidney 293T cells. The culture media containing recombinant lentiviruses were harvested 48 and 72 hours after transfection, filtered through a 0.45-µm filter, and transduced into SH-SY5Y cells using 8 to 10 µg/ml polybrene. Transduced cell cultures were incubated with 1 to 2 µg/ml puromycin at 48 to 72 hours post transduction. Puromycin-resistant colonies were selected with the same concentration of puromycin for 2 to 3 weeks.

### Chemicals

Anisomycin (Sigma, A9789), azidohomoalanine (AHA) (Invitrogen, C10102), cycloheximide (CHX) (Sigma, 01810), harringtonine (Cayman, 15361), homoharringtonine (Sigma, SML1091), fluor 488-alkyne (Sigma, 761621), MG-132 (Enzo, BML-PI102), nilotinib (Apexbio, A8232), and sorafenib (Apexbio, A3009) were dissolved in DMSO and stored at −20°C before use. Puromycin dihydrochloride (Sigma-Aldrich, P8833) was dissolved in PBS and stored at −20°C before use. Phos-tag acrylamide (Fujifilm, AAL-107) was dissolved in 3% (v/v) methanol and stored at 4°C before use.

### Plasmids and siRNAs

pmGFP-P2A-K0-P2A-RFP (Addgene plasmid #105686) and pmGFP-P2A-K20-P2A-RFP (Addgene plasmid #105688) were gifts from Ramanujan Hegde (Juszkiewicz and Hegde, 2017). pCDH-EF1-LTN1 and pCDH-EF1-LTN1ΔRING were gifts from Toshifumi Inada (Udagawa et al., 2021). *DNAJC2* cDNA was PCR-amplified and subcloned into pcDNA3.1 to overexpress untagged DNAJC2. Overlapping PCRs introduced 7 nucleotide mutations into wild-type *DNAJC2* cDNA to generate a siRNA-resistant clone. Oligonucleotides corresponding to the huntingtin uORF (Aviner et al., 2024) were subcloned into pmGFP-P2A-K0-P2A-RFP or pmGFP-P2A-K20-P2A-RFP. Oligonucleotides encoding either the wild-type C-terminal sequence of human unspliced XBP1 (XBP1u) or one of 3 XBP1u stalling sequences (wild-type, S255A, or W256A), each followed by 4 additional lysine residues (Juszkiewicz et al., 2020a), were subcloned into pmGFP-P2A-K0-P2A-RFP to generate XBP1 translation reporters. pLentiPGK Puro DEST JNKKTRClover (Addgene plasmid #59151) was a gift from Markus Covert (Regot et al., 2014). pCMV-P-RAN-RFP6 was a gift from Joshua Sanes (Addgene plasmid #106411) (Yamagata and Sanes, 2018). lentiCRISPR v2 was a gift from Feng Zhang (Addgene plasmid #52961) (Sanjana et al., 2014). Oligonucleotides corresponding to sgRNA for *ZAK* (5’-ATG GAT ATC ACA GGA CAA GG-3’) (Wu et al., 2020) were subcloned into lentiCRISPR v2. Control and gene-specific siRNAs were synthesized (Genolution and Bioneer) as listed in Supplementary Table S1.

### Flow Cytometry

Flow cytometry analysis of translation reporters was conducted as described previously (Park et al., 2021b) with minor modifications. Reporter-transfected cells were harvested by trypsinization at 48 hours post transfection, briefly washed with ice-cold PBS twice, and resuspended in PBS for flow cytometry analysis. Fluorescence intensities of GFP and mCherry were measured in individual cells using LSRFortessa (BD Biosciences) and analyzed in 20,000 GFP-positive cells using FlowJo software.

### Immunoblotting

Transfected cells were harvested and lysed in a lysis buffer [25 mM Tris-Cl, pH 7.5, 300 mM NaCl, 10% glycerol, 1 mM EDTA, and 0.5% NP-40 (v/v)] supplemented with 1 mM DTT and 1 mM PMSF. For puromycin labeling of nascent polypeptides, cells were first treated with 2 µM harringtonine for the indicated time and then incubated with 7.5 μg/ml puromycin for 15 minutes at 37°C before harvest. Protein concentrations were determined using Pierce BCA Protein Assay Kit (Thermo Fisher Scientific). Cell lysates were mixed with Laemmli sample buffer and boiled for 5 minutes. Equal amounts of protein samples were loaded into individual wells of multiple acrylamide gels in parallel to avoid complications from stripping and reprobing, resolved by SDS-PAGE, and transferred to the nitrocellulose membranes by wet electroblotting (Bio-Rad). For Phos-tag gel electrophoresis, Laemmli sample buffer without EDTA was used. SDS-PAGE gels were supplemented with 50 µM Phos-tag acrylamide (Fujifilm) and 100 µM MnCl_2_. Before membrane transfer, the gels were washed for 10 minutes in transfer buffer containing 1 mM EDTA, followed by a second wash in transfer buffer without EDTA with gentle agitation. Transferred membranes were blocked with 5% (w/v) nonfat dried milk or bovine serum albumin in TBS containing 0.05% (v/v) Tween-20 (TBS-T) for 30 minutes at room temperature, then incubated with primary antibodies at 4°C overnight. After washing 4 times for 5 minutes with TBS-T, membranes were further incubated with HRP-conjugated secondary antibodies (Thermo Fisher Scientific) at room temperature for 1 hour, then washed 3 times for 5 minutes with TBS-T. The chemiluminescence from immunoblots was detected using iBright1500 (Thermo Fisher Scientific) with Clarity Western (Bio-Rad) or WestGlow FEMTO (Biomax) ECL substrates. The RANbody for mCherry detection was generated in 293T cells by transfecting with pCMV-P-RAN-RFP6. Transfected cell cultures were incubated for 48 hours before harvesting the culture medium. The RANbody-containing medium was filtered with a 0.45-µm filter, supplemented with 0.1% (v/v) ProClin 150 (Sigma), and stored at 4°C before use. The RANbody was diluted at 1:2 in PBS-T containing 5% (v/v) nonfat dried milk for primary antibody incubation. Protein band intensities in immunoblots were quantified using ImageJ/Fiji (NIH). Antibodies used in this study are listed in Supplementary Table S1.

### Polysome Profiling

Polysome profiling using sucrose density gradient was performed as described previously with some modifications (Ingolia et al., 2011, Stoneley and Harvey, 2022). Wild-type and *ZNF598*^KO^ SH-SY5Y cells on 100-mm culture dishes were harvested 72 hours after siRNA transfection. For harringtonine runoff, cells were treated with 4 µM harringtonine for the indicated time and then incubated with 100 µg/ml CHX for 5 minutes. For the collision-induced ribosome disassembly assay, cells were treated with 500 nM of anisomycin for the indicated time. Cells were washed with ice-cold PBS containing 100 µg/ml CHX, harvested by centrifugation, and lysed in a polysome lysis buffer [25 mM Tris-Cl, pH 7.5, 150 mM KCl, 5 mM MgCl_2_, and 0.5% NP-40 (v/v)] supplemented with 1 mM DTT and 100 µg/ml CHX for 15 minutes on ice. After centrifugation at 13,500 rpm for 15 minutes at 4°C, absorbance at 260 nm (OD260) was measured in soluble extracts using a NanoDrop One spectrophotometer (Thermo Fisher Scientific). Normalized cell extracts were layered onto 10 to 50% sucrose density gradients containing 25 mM Tris-Cl, pH 7.5, 150 mM KCl, 5 mM MgCl_2_, and 100 µg/ml CHX. Gradient samples were centrifuged at 36,000 rpm for 3 hours at 4°C using a SW-41Ti rotor (Beckman). Absorbance at 250 nm was continuously measured during their fractionation using a BR-188 density gradient fractionation system (Brandel) or an RDn chromatography detector (Reach devices). The relative abundance of ribosomal peaks was quantified by measuring the area under the curve using the Peak Analyzer tool in OriginPro 2020 (OriginLab Corporation), as described previously (Biever et al., 2020).

### Quantitative Transcript Analysis

Total RNA was isolated from SH-SY5Y cells using a TaKaRa MiniBEST Universal RNA Extraction Kit (TAKARA). Two μg of each RNA sample was subjected to cDNA synthesis using M-MLV RT reverse transcriptase (Promega) according to the manufacturer’s instructions. Diluted cDNA samples were used for real-time PCR by QuantStudio 1 Real-Time PCR systems (Applied Biosystems) using Primer Q-Master mix (Genetbio) and gene-specific primer pairs. *GAPDH* served as a normalizing transcript to calculate relative expression levels of RSR target genes. Primers used in this study are listed in Supplementary Table S1.

### Live-Cell Imaging of JNK-KTR

Wild-type or *ZNF598*^KO^ cells stably expressing the JNK-KTR reporter were seeded onto the glass-bottom dish (SPL). siRNA-transfected cells were treated with 62.5 nM anisomycin at 72 hours post transfection and further incubated at 37°C and 5% CO_2_ for live-cell imaging using a confocal microscope (LSM780) with a 40X oil immersion objective. Time-series images were taken every 20 seconds for 15 minutes after anisomycin treatment. Where indicated, DMSO (vehicle control), 10 µM sorafenib, or 10 µM nilotinib was added to the live-cell cultures 15 minutes before anisomycin treatment. For quantification of JNK activation, both cytoplasmic and nuclear fluorescence of the JNK-KTR reporter was measured in each cell per 20-sec frame, and the ratio of cytoplasmic-to-nuclear fluorescence intensities (ie, C/N ratio) was traced over a 15-minute recording.

### Click Chemistry Labeling and Confocal Microscopy

For nascent polypeptide labeling, SH-SY5Y cells cultured on coverslips were washed with prewarmed PBS, equilibrated with methionine-free medium for 30 minutes, and then incubated with 1 mM azidohomoalanine (AHA) for the indicated times at 37°C incubator. After AHA labeling, cells were washed with ice-cold PBS and fixed in PBS containing 3.7% (w/v) formaldehyde for 15 minutes at room temperature, and then permeabilized in PBS containing 0.1% (v/v) Triton X-100 (PBS-T) at 4°C for 15 minutes. For the click chemistry reaction, cells were washed with PBS containing 1% (w/v) BSA and then incubated with Click-iT cell reaction cocktail (Thermo Fisher Scientific) and 1 mM Fluor 488-alkyne (Sigma) for 30 minutes at room temperature with gentle shaking. After washing with 1% (w/v) BSA and staining the nuclei with Hoechst 33342 (Thermo Fisher Scientific) for 5 minutes at room temperature, the coverslips were mounted onto slides with VECTASHIELD PLUS (Vector Laboratories). Confocal images were acquired using a Nikon A1 confocal microscope (60X objective) with NIS-Elements software (Nikon). All image quantifications and analyses were performed with ImageJ/Fiji (NIH). For quantification of nascent polypeptides, the fluorescence intensity was measured from the total area of individual AHA-labeled cells. Relative levels of nascent polypeptides were then calculated by normalizing the average fluorescence intensity of each condition to that of control cells at 60 minutes.

### Statistical Analysis

All statistical analyses were conducted using Prism 10 (GraphPad Software) or R (ver. 3.6.1) with ARTool library as described previously (Park et al., 2023). Normality and equal variance in the given datasets were determined by Shapiro-Wilk and Brown-Forsythe tests, respectively. Datasets with low sample numbers (*n* < 14) or parametric datasets with equal variance were analyzed by unpaired *t* test or ordinary ANOVA with Tukey’s multiple comparisons test. Nonparametric datasets were analyzed by aligned ranks transformation (ART) ANOVA with Wilcoxon rank-sum test. All numeric data generated in this study were included in Supplementary Table S2. Sample sizes, statistical methods used, and *P* values obtained from individual tests were summarized in Supplementary Table S3 and appropriately noted in the corresponding figure legends.

## RESULTS

### The Ribosomal Chaperone DNAJC2 Facilitates RQC-Sensitive Poly-A Translation

Dual-fluorescence translation reporters have been well established to measure cellular RQC activities (Juszkiewicz and Hegde, 2017) (Fig. 1A). In these reporters, upstream GFP serves as an internal reference for reporter expression, whereas downstream mCherry reports translation through intervening test sequences. Flow cytometry allows quantitative measurement of both fluorescence signals in individual cells, and a lower mCherry-to-GFP ratio is thus consistent with increased translational stalling on the given test sequence. The RQC-sensitive poly-A translation reporter encoding polylysine repeats [ie, K(AAA)_20_] shows a lower mCherry-to-GFP ratio than the control K(AAA)_0_ reporter. The reduced downstream expression likely reflects RQC-mediated disassembly of collided ribosomes during poly-A translation. To identify trans-acting factors that regulate collision-prone poly-A translation, we performed an RNA interference (RNAi) screen targeting polynucleotide-binding proteins and translation-coupled quality control factors. These analyses revealed that depletion of ZNF598 or small-subunit ribosomal proteins, including RPS3, RPS10, and RPS20, increased the mCherry-to-GFP ratio (Fig 1B). ZNF598 ubiquitinates RPS10 and RPS20 upon ribosome collision to initiate RQC (Juszkiewicz and Hegde, 2017, Sundaramoorthy and Leonard, 2017). Depleting these ribosomal proteins may suppress ZNF598-dependent RQC initiation and subsequent disassembly of collided ribosomes during K(AAA)_20_ translation. This could promote translational readthrough of the ribosome-stalling sequence, thereby increasing downstream mCherry expression relative to GFP. More generally, RPS depletion may reduce productive translation initiation and ribosome loading. Such effects could decrease ribosome collisions and RQC activation during K(AAA)_20_ translation, contributing to the increased mCherry-to-GFP ratio observed upon RPS depletion (Fig. 1B). Among the candidate genes tested, depletion of the RAC component DNAJC2 most strongly exaggerated the translational stalling on the poly-A tract but had negligible effects on the control reporters K(AAA)_0_ and K(AAA)_5_ (Fig. 1B, C). Genomic deletion of the collision-sensing RQC factor ZNF598 consistently increased the ratio of mCherry-to-GFP expression from the K(AAA)_20_ reporter (Juszkiewicz and Hegde, 2017, Park et al., 2021b) and blunted the DNAJC2-depletion phenotypes (Fig. 1D). These results functionally link DNAJC2 activity to ZNF598-sensitive translational stalling.

**Fig. 1:**
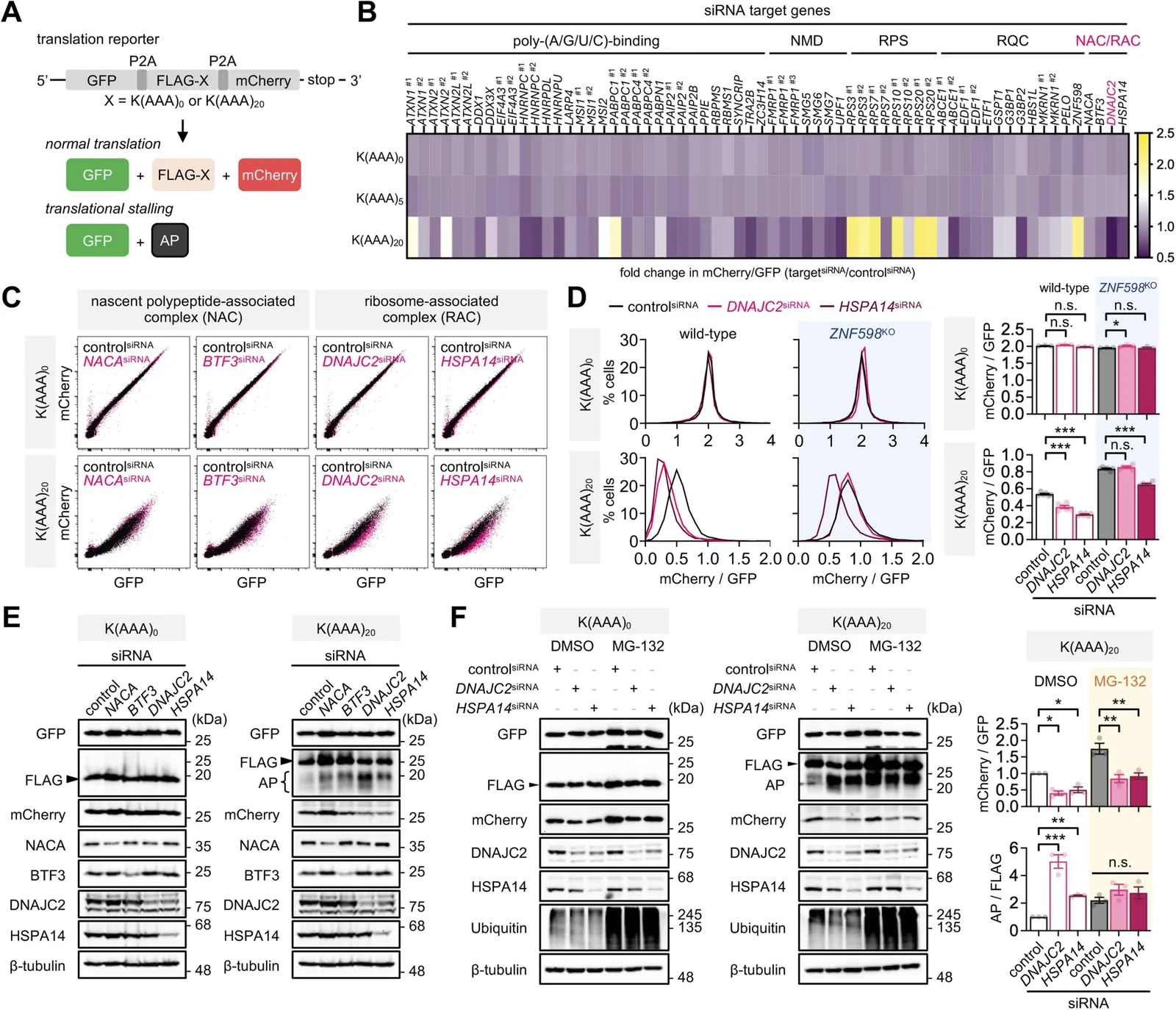
*Depletion of the ribosomal chaperone DNAJC2 enhances translational stalling on the RQC-sensitive K(AAA)_20_ reporter and arrested product accumulation.* (**A**) Schematic diagram of the dual-fluorescence reporter for measuring translational stalling and RQC activity. K(AAA)_0_, control reporter; K(AAA)_20_, RQC-sensitive poly-A translation reporter that encodes 20 repeats of the lysine codon; P2A, a 2A peptide that induces ribosomal skipping during translation; AP, translationally arrested products. (**B**) A siRNA-based screen identifies DNAJC2 as an enhancer of poly-A translation. The median ratio of mCherry-to-GFP expression in target gene siRNA-transfected cells was normalized to that in control siRNA-transfected cells. The relative reporter expression was then displayed as a heatmap. SH-SY5Y cells were transfected with the reporter plasmids 24 hours after each siRNA transfection and further incubated for 48 hours. The fluorescence reporter expression was analyzed in individual cells using flow cytometry. (**C**) Depletion of the RAC (DNAJC2 and HSPA14) but not NAC components (NACA and BTF3) enhances translational stalling on the RQC-sensitive poly-A tract. Reporter expression in individual cells was visualized by dot plots (*n* = 15,000-20,000 cells). Black dots, control siRNA-transfected cells; pink dots, target gene siRNA-transfected cells. (**D**) *ZNF598* deletion blunts RAC-depletion effects on the translation-stalling K(AAA)_20_ reporter. Histograms depict the percentages of cells displaying the given ratio of mCherry-to-GFP expression from each translation reporter (left). The median ratio values were determined from independent experiments [*n* = 3 for K(AAA)_0_ reporter; *n* = 6 for K(AAA)_20_ reporter] and compared among different genetic backgrounds (right). Two-way ANOVA detected significant interaction effects between RAC depletion and *ZNF598* deletion (*ZNF598*^KO^) on the K(AAA)_20_ reporter expression (*P* < .001 for *DNAJC2*; *P* = .005 for *HSPA14*). Data represent mean +/- SEM. n.s., not significant; *P < .05, ***P < .001 as determined by Tukey’s multiple comparisons test. (**E**) RAC depletion elevates K(AAA)_20_-derived arrested product levels. Reporter protein expression was analyzed by immunoblotting of total cell lysates. Arrowheads, FLAG-tagged full-length products from K(AAA)_0_ or K(AAA)_20_ translation. (**F**) Proteasomal inhibition masks RAC-depletion effects on K(AAA)_20_-derived arrested product expression (AP/FLAG) but not on the poly-A translation stalling (mCherry/GFP). Transfected cells were incubated with the proteasome inhibitor (20 µM MG-132) for 12 h before immunoblotting analyses. Reporter protein expression was quantified by band intensities in immunoblots. Data represent mean +/- SEM (*n* = 3). n.s., not significant; *P < .05, **P < .01, ***P < .001 as determined by 2-way ANOVA, Tukey’s multiple comparisons test.

To complement the flow cytometry analyses, we next examined the translation reporter products by immunoblotting. In particular, anti-FLAG immunoblotting distinguishes the full-length FLAG-tagged middle reporter product (FLAG) from shorter translationally arrested products (AP) to quantify their relative abundance (ie, AP/FLAG ratio). These analyses confirmed that DNAJC2 depletion suppressed poly-A readthrough during translation and elevated the levels of translationally arrested products from the K(AAA)_20_ reporter (Fig. 1E). The *DNAJC2* phenotypes were consistently observed with independent small interfering RNAs (siRNAs) but not by transfection reagents per se, arguing against off-target effects (Supplementary Fig. S1A, B). Furthermore, overexpression of a siRNA-resistant DNAJC2 cDNA partially rescued the arrested product phenotypes in DNAJC2-depleted cells (Supplementary Fig. S1C). The abundance of translationally arrested products is a steady-state measure reflecting both their formation and clearance, rather than a direct measure of the translational stalling rate. Treatment with the proteasome inhibitor MG-132 masked *DNAJC2* effects on the K(AAA)_20_-derived arrested product levels but not on the translational stalling (Fig. 1F and Supplementary Fig. S1D), suggesting that proteasome-dependent turnover influences the steady-state arrested product abundance downstream of *DNAJC2*. RNAi-mediated depletion of the other RAC component HSPA14, but not the NAC components (ie, NACA or BTF3), phenocopied *DNAJC2* effects on the K(AAA)_20_ translation (Fig. 1B-F). In fact, HSPA14-targeting siRNAs co-depleted DNAJC2 but not vice versa (Fig. 1E, F; Supplementary Fig. S2A), likely indicating that HSPA14 stabilizes DNAJC2 upon RAC assembly (Wu et al., 2021, Yi et al., 2020). Nonetheless, HSPA14 depletion effects on the K(AAA)_20_-derived arrested products were modest compared with DNAJC2 depletion (Fig. 1E, F; Supplementary Fig. S2A). We also found that *ZNF598* deletion did not completely mask translational stalling of the K(AAA)_20_ reporter in HSPA14-depleted cells (Fig. 1D; Supplementary Fig. S2B, C), suggesting that *HSPA14* effects on poly-A readthrough may involve additional contributions from *ZNF598*-independent mechanisms. Previous studies have similarly reported distinct depletion phenotypes and biochemical interactions of individual RAC components (Meller and Coombs, 2020, Wu et al., 2021, Yi et al., 2020). Collectively, these results strongly support the idea that DNAJC2 facilitates ZNF598-sensitive poly-A translation, and we accordingly focused on *DNAJC2* phenotypes in subsequent analyses.

### DNAJC2 Depletion Enhances the Accumulation of Translationally Arrested Products From the Endogenous XBP1u Stalling Sequence

To determine whether DNAJC2-depletion phenotypes extend beyond the poly-A tract, we examined reporters containing a stalling sequence from human unspliced XBP1 (XBP1u). The XBP1u nascent chain induces translational pausing, which facilitates the targeting of its mRNA to the endoplasmic reticulum and subsequent IRE1-mediated processing (Yanagitani et al., 2011). XBP1u has also been identified as an endogenous RQC substrate based on ribosome collisions and ZNF598- and LTN1-dependent quality control (Han et al., 2020). We first inserted the wild-type XBP1u C-terminal sequence (residues 226-261) into the dual-fluorescence reporter (Supplementary Fig. S3A). DNAJC2 depletion increased the levels of arrested products relative to full-length FLAG-tagged products from the wild-type XBP1u reporter, but not from the K(AAA)_0_ control reporter (Supplementary Fig. S3B). We further examined translation reporters containing the wild-type XBP1u stalling sequence or its stronger-stalling S255A and weaker-stalling W256A variants, each followed by 4 additional lysine residues (Juszkiewicz et al., 2020a) (Supplementary Fig. S3A). DNAJC2 depletion consistently increased arrested product accumulation from the wild-type and S255A reporters, whereas no significant increase was observed for the W256A reporter (Supplementary Fig. S3C). The mCherry-to-GFP ratios were not significantly altered by DNAJC2 depletion in either reporter series (Supplementary Fig. S3B, C). These results demonstrate that DNAJC2-dependent arrested product accumulation is not restricted to artificial poly-A stalling but can also be observed with the endogenous XBP1u stalling sequence. They further suggest that *DNAJC2* effects may depend on the stalling properties of the reporter sequence.

### DNAJC2 Limits Collision-Prone Translational Burden Onto the Canonical RQC Pathway

To further elucidate how DNAJC2 supports RQC-sensitive poly-A translation, we compared DNAJC2-depletion effects on the K(AAA)_20_ reporter expression across genetic backgrounds with distinct RQC defects. *ZNF598* deletion suppressed arrested product generation from the K(AAA)_20_ reporter, silencing *DNAJC2* effects on poly-A translation stalling and arrested product levels (Fig. 2A, B). In contrast, *LTN1* deletion showed synergistic effects with DNAJC2 depletion on arrested product accumulation. Overexpression of wild-type LTN1 but not a deletion mutant defective in E3 ligase activity reduced the K(AAA)_20_-derived arrested product levels comparably in control and DNAJC2-depleted cells (Supplementary Fig. S4A, B). It is thus unlikely that DNAJC2 depletion impairs LTN1 function per se. *NEMF*-deleted cells displayed smaller arrested product species from the K(AAA)_20_ reporter (Fig. 2A), possibly due to a lack of NEMF-dependent CAT-tailing on the RQC substrates (Kostova et al., 2017, Shen et al., 2015, Tesina and Ebine, 2023, Udagawa et al., 2021). More importantly, *NEMF* deletion blunted *DNAJC2* effects on the K(AAA)_20_-derived arrested product accumulation (Fig. 2A, B). In contrast, more quantitative analyses of reporter expression by flow cytometry showed that *NEMF* deletion did not significantly affect DNAJC2-sensitive translational stalling on the poly-A tract (Supplementary Fig. S4C, D). These distinct effects are consistent with NEMF acting downstream of ribosome disassembly to regulate arrested product processing and accumulation.

**Fig. 2:**
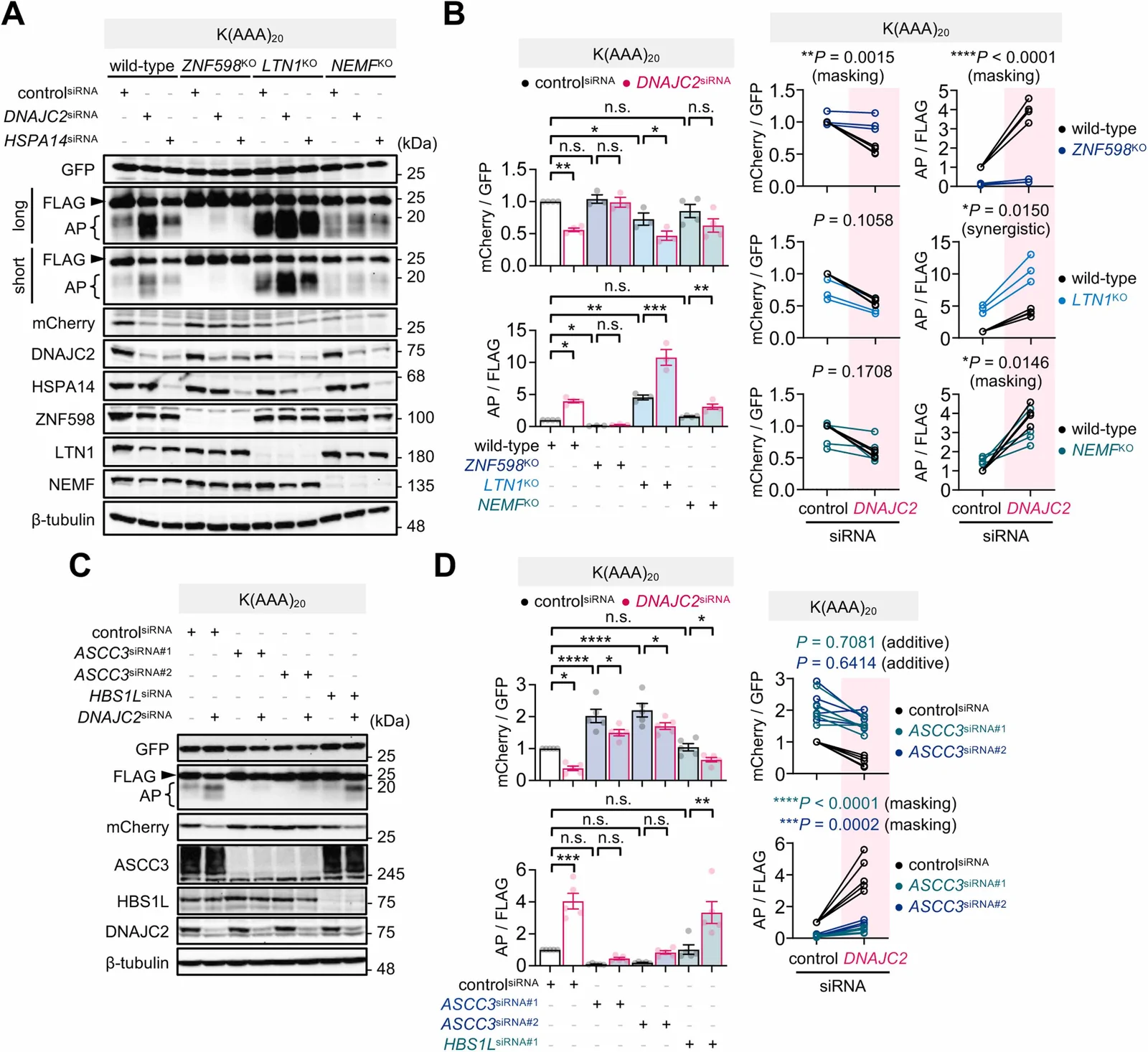
*DNAJC2 acts upstream of the canonical RQC pathway to reduce collision-prone translation intermediates.* (**A, B**) *ZNF598* deletion masks DNAJC2-depletion effects on the RQC-sensitive K(AAA)_20_ reporter expression. Reporter protein expression in different genetic backgrounds was assessed by immunoblotting, similarly as in Figure 1. Two-way ANOVA detected significant interaction effects of DNAJC2 depletion with *ZNF598* deletion on translational stalling (mCherry/GFP) and arrested product generation (AP/FLAG) during the K(AAA)_20_ reporter expression (B). The interaction *P* values were shown at the top of each graph. Data represent mean +/- SEM (*n* = 4 for wild-type and *NEMF*^KO^; *n* = 3 for *ZNF598*^KO^ and *LTN1*^KO^); data points connected by lines represent paired measurements from the same experiment. n.s., not significant; *P< .05, **P < .01, ***P < .001 as determined by Tukey’s multiple comparisons test. (**C, D**) Depletion of ASCC3 but not of HBS1L suppresses *DNAJC2* effects on K(AAA)_20_-derived arrested product generation. Two-way ANOVA detected significant interaction effects of *DNAJC2* and *ASCC3* on relative arrested product levels to full-length K(AAA)_20_ proteins (AP/FLAG) (D). The interaction *P* values were shown at the top of each graph (green, *ASCC3*^siRNA#1^; blue, *ASCC3*^siRNA#2^). Data represent mean +/- SEM (*n* = 5); data points connected by lines represent paired measurements from the same experiment. n.s., not significant; *P < .05, **P < .01, ***P< .001, ****P < .0001 as determined by Tukey’s multiple comparisons test.

The RQT complex is recruited to ZNF598-primed collided ribosomes for stalled-ribosome disassembly, and the RNA helicase ASCC3 serves as a key component of the RQT complex in this process (Hashimoto et al., 2020, Juszkiewicz and Speldewinde, 2020, Matsuo et al., 2017, Stoneley and Harvey, 2022). To determine whether *DNAJC2* effects on poly-A translation require RQT complex-mediated ribosome disassembly, we performed an additional epistasis analysis of *DNAJC2* and *ASCC3* on the reporter expression. ASCC3 depletion per se alleviated translation stalling on the K(AAA)_20_ reporter and suppressed its arrested product generation (Fig. 2C, D; Supplementary Fig. S5A). Moreover, ASCC3 deficiency potently suppressed arrested product generation in DNAJC2-depleted cells while weakening their translational stalling on the K(AAA)_20_ reporter. The *ASCC3* effects were relatively specific since RNAi of the no-go decay ribosome rescue factor HBS1L (Pisareva et al., 2011, Shoemaker et al., 2010) did not affect the *DNAJC2* phenotypes (Fig. 2C, D; Supplementary Fig. S5B). This series of epistasis analyses thus defines DNAJC2 as an upstream regulator of the canonical RQC pathway. These findings suggest that DNAJC2 may titrate ZNF598-primed ribosomes and their RQT-mediated dissociation during collision-prone translation. This may reduce the generation of arrested nascent chain substrates that are subsequently processed by NEMF and targeted for LTN1-dependent proteasomal clearance.

### DNAJC2 Titrates Ribosome Collision-Induced RSR Activation

We asked whether DNAJC2 could shape translation stress responses to global ribosome collisions beyond RQC-sensitive poly-A translation. Translation elongation inhibitors and other ribotoxic cues induce ribosome stalling and collisions, activating the dedicated protein kinase ZAKα (mitogen-activated protein kinase kinase kinase 20, MAP3K20) and initiating the ribotoxic stress response (RSR) (Iordanov et al., 1997, Vind et al., 2020, Wu and Peterson, 2020). The RSR involves a MAPK signaling cascade, including phosphorylation of ZAKα and the downstream stress-activated protein kinases (SAPKs) c-Jun N-terminal kinase (JNK; MAPK8) and p38 (MAPK14). Consistent with previous observations (Vind et al., 2020, Wu and Peterson, 2020), wild-type cells displayed a specific window of anisomycin concentrations that induced dose-dependent phosphorylation of the RSR-relevant kinases, as assessed by Phos-tag gel electrophoresis or immunoblotting with phosphorylation-specific antibodies (Fig. 3A). DNAJC2 depletion shifted the effective window toward lower anisomycin concentrations, facilitating anisomycin-induced RSR. The sensitized RSR in DNAJC2-depleted cells was consistently observed with independent RNAi transgenes, excluding off-target effects (Supplementary Fig. S6A). To better quantify the RSR kinetics, we employed a fluorescence kinase translocation reporter for JNK activity (JNK-KTR) (Miura et al., 2018, Regot et al., 2014). Upon JNK activation, the JNK-KTR reporter is phosphorylated and translocated to the cytoplasm (Fig. 3B). We therefore monitored the ratio of cytoplasmic-to-nuclear fluorescence in individual cells over time by live-cell imaging. This approach indeed validated that DNAJC2 depletion facilitated JNK activation upon anisomycin treatment, whereas *ZNF598* deletion substantially blunted the *DNAJC2* phenotypes (Fig. 3B, C; Supplementary Fig. S6B). ZAK-dependent activation of the JNK-KTR sensor was validated by general ZAK inhibitors or ZAK depletion that silenced the reporter activation in either wild-type or DNAJC2-depleted cells (Fig. 3D and Supplementary Fig. S6B). Finally, quantitative analyses of the RSR-induced gene transcripts confirmed that DNAJC2 depletion exaggerated the transcriptional response to *ZAK*-dependent RSR (Sinha et al., 2020, Subramanian et al., 2023, Vind et al., 2020, Vind et al., 2024), whereas genomic deletions of *ZNF598* or *ZAK* suppressed the *DNAJC2* phenotypes (Fig. 3E). These data suggest that DNAJC2 can limit both transcript-specific and global ribosome collisions to titrate the threshold for downstream activation of RQC and translation stress signaling pathways, respectively.

**Fig. 3:**
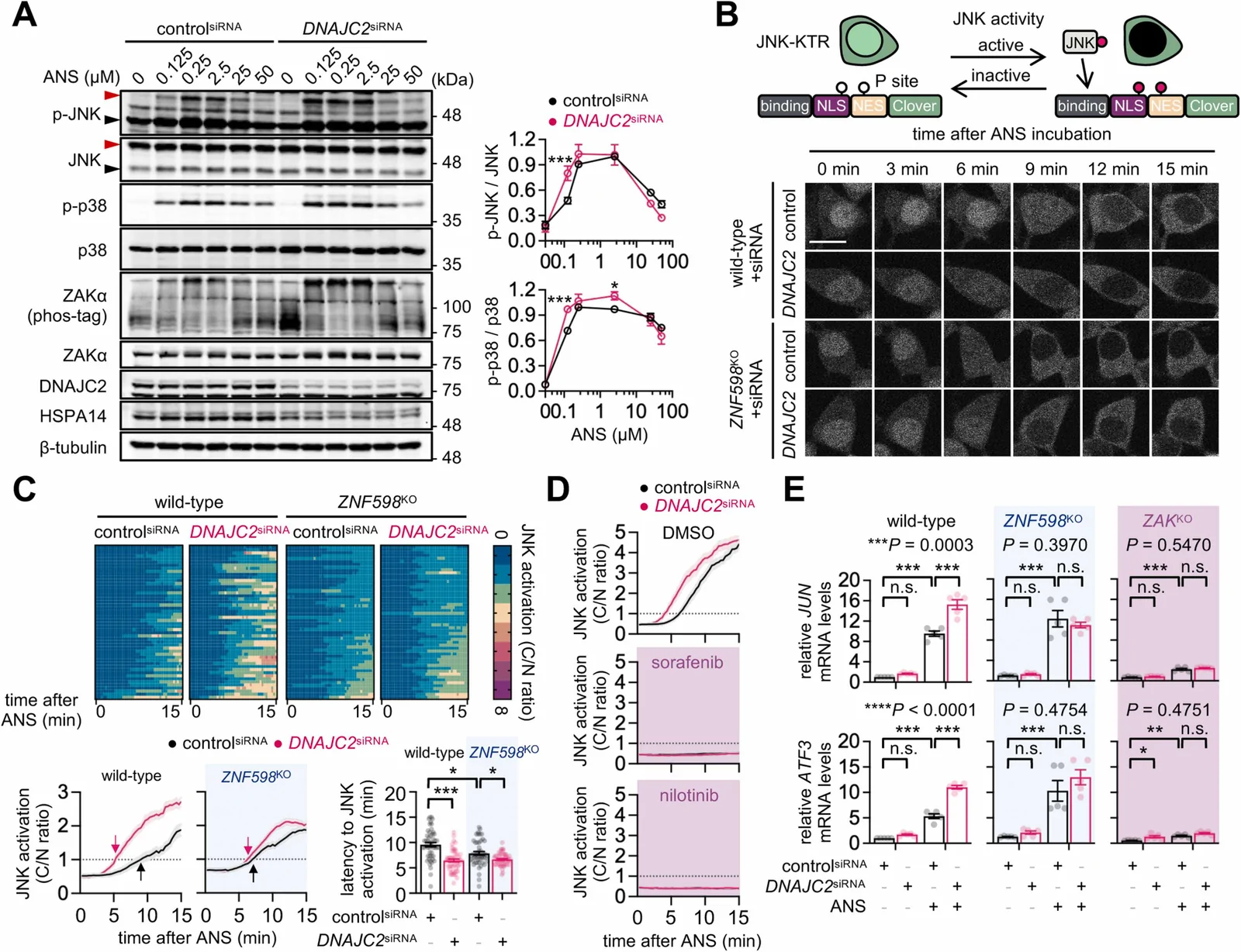
*DNAJC2 titrates ribosome collision-induced RSR activation.* (**A**) DNAJC2 depletion facilitates RSR activation upon anisomycin-induced ribosome collision. siRNA-transfected SH-SY5Y cells were incubated with increasing amounts of anisomycin or vehicle control (DMSO) for 15 minutes before harvest. Total cell lysates were analyzed by immunoblotting similarly as in Figure 1. Phosphorylated proteins were detected using phospho-specific antibodies (p-JNK, p-p38) or Phos-tag gel electrophoresis (ZAKα). Relative phosphorylation levels of the stress-activated kinases (JNK/p54 and p38) were quantified by normalizing to the maximum ratio value in control lanes (set as 1). Data represent mean +/- SEM (*n* = 4 for p-JNK/JNK; *n* = 3 for p-p38/p38). *P< .05, ***P < .001 to control siRNA at the given anisomycin concentrations as determined by 2-way ANOVA, Tukey’s multiple comparisons test. Red arrowheads, p54 isoform; black arrowheads, p46 isoform. (**B**) Schematic diagram of the JNK-KTR sensor and the underlying principle (top). JNK activation induces the phosphorylation-dependent translocation of the KTR sensor from the nucleus (N) to the cytoplasm (C), increasing the C/N ratio of the fluorescence intensity. Representative live-cell imaging of the JNK-KTR sensor in different genetic conditions upon anisomycin treatment (bottom). Scale bar = 10 µm. (**C**) DNAJC2 depletion facilitates anisomycin-induced JNK-KTR activation in a *ZNF598*-dependent manner. Fluorescence from the JNK-KTR sensor was recorded by live-cell imaging. The C/N ratio of the fluorescence intensity was calculated in individual cells over time and displayed as a heatmap (top) or on average (bottom). Arrows indicate time points (latency to JNK activation) when the C/N ratio of the JNK-KTR signals goes above a threshold (dotted lines; set as 1). Aligned ranks transformation (ART) 2-way ANOVA detected significant interaction effects of DNAJC2 depletion and *ZNF598* deletion on the latency to JNK-KTR activation (*P*= .0180). Data represent mean +/- SEM (*n* = 49–52). *P < 0.05, ***P < .001 as determined by the Wilcoxon rank-sum test. (**D**) The ZAK inhibitors sorafenib and nilotinib suppress anisomycin-induced JNK-KTR activation in both control- and DNAJC2-depleted cells. siRNA-transfected cells were preincubated with 10 µM sorafenib or nilotinib 15 min before anisomycin treatment. Data represent mean +/- SEM (*n* = 28–30). (**E**) DNAJC2 depletion enhances anisomycin-induced expression of RSR target genes in a *ZNF598*- or *ZAK*-dependent manner. siRNA-transfected cells were incubated with 100 nM anisomycin 1 h before harvest. Each transcript was quantified using real-time PCR with gene-specific primer pairs. Relative mRNA levels were calculated by normalizing to those in control siRNA-transfected cells treated with DMSO (vehicle). Two-way ANOVA detected significant interaction effects of DNAJC2 depletion and anisomycin treatment on *JUN* or *ATF3* mRNA levels (*P* = .0003 or *P* < .0001, respectively) in wild-type but not in *ZNF598*- or *ZAK*-deleted cells. Data represent mean +/- SEM (*n* = 5). n.s., not significant; *P< .05, **P < .01, ***P < .001 as determined by Tukey’s multiple comparisons test. ANS denotes anisomycin in the figure panels.

### DNAJC2 Restrains Global Translational Flux to Reduce RQC Burden During Collision-Prone Translation

To understand the roles of DNAJC2 in global ribosome collisions, we compared general translation activities in control vs DNAJC2-depleted cells. When nascent polypeptides were quantitatively assessed in individual cells by incorporation of the amino acid analog azidohomoalanine (AHA) (Beatty and Tirrell, 2008, Dieterich et al., 2010, Roche et al., 2009, Tom Dieck et al., 2012), DNAJC2-depleted cells showed more robust AHA labeling than control cells (Fig. 4A), consistent with a previous observation (Wu et al., 2021). Sucrose density gradient profiling showed a higher polysome-to-monosome ratio at baseline in DNAJC2-depleted cells (Fig. 4B). This baseline ratio alone does not establish the elongation rate because it reflects both ribosome loading and the contributions of stalled and actively translating ribosomes. We therefore examined ribosome runoff after harringtonine treatment. Harringtonine prevents new ribosomes from entering productive elongation, while ribosomes already elongating can complete translation and leave the mRNA (Ingolia et al., 2011). The rapid decline in the polysome-to-monosome ratio after treatment showed that the elevated polysome population was actively translating (Fig. 4B). To assess elongation kinetics more directly, we measured puromycin incorporation after increasing periods of harringtonine treatment (Arguello et al., 2018) (Fig. 4C–E). As elongating ribosomes run off, fewer nascent chains remain available for puromycin labeling. DNAJC2-depleted cells showed a faster decline in puromycin-labeled polypeptides following harringtonine treatment, supporting the conclusion that DNAJC2 depletion accelerates translation elongation (Fig. 4D, E). Together, these results indicate that DNAJC2 depletion enhances global translation output, at least in part, by increasing the rate of translation elongation.

**Fig. 4:**
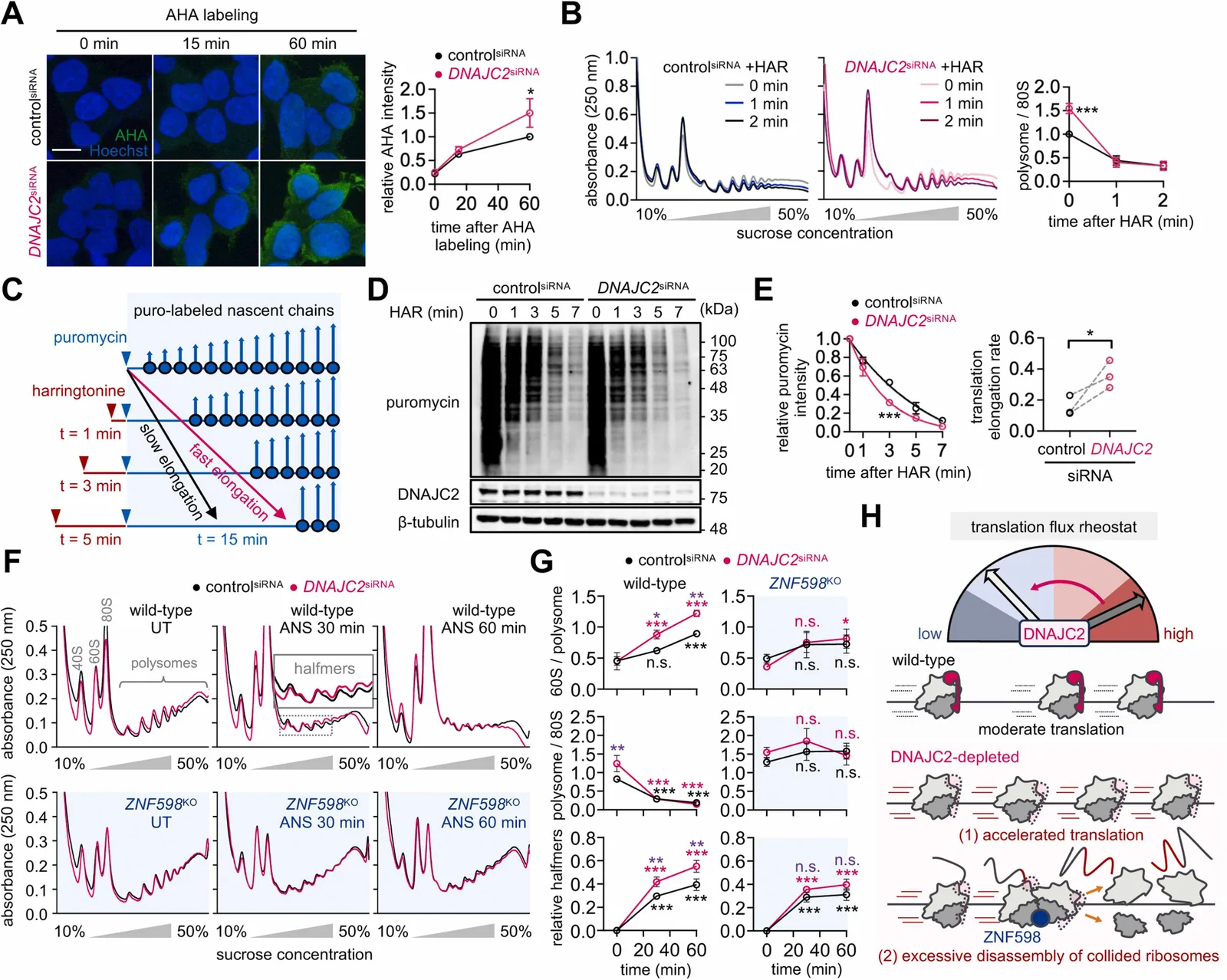
*DNAJC2 restrains global translational flux to reduce RQC burden during collision-prone translation.* (**A**) DNAJC2-depleted cells incorporate the amino acid analog azidohomoalanine (AHA) more robustly into nascent polypeptides. siRNA-transfected SH-SY5Y cells were incubated in a methionine-free medium for 30 minutes before AHA labeling (1 mM AHA, 0/15/60 minutes). AHA-labeled proteins were probed with Fluor 488-alkyne using a Click-iT cell reaction cocktail (green) and visualized using confocal microscopy. Relative AHA intensity was calculated by normalizing to the averaged fluorescence measured in control cells at 60 minutes (*n* = 30 cells, set as 1). Data represent mean +/- SEM (*n* = 3 experiments). *P< .05 to control cells (60 minutes) as determined by 2-way ANOVA, Tukey’s multiple comparisons test. Scale bar = 10 µm. (**B**) DNAJC2-depleted cells exhibit high levels of actively translating ribosomes. siRNA-transfected SH-SY5Y cells were incubated with the translation initiation inhibitor harringtonine (4 µM, 0/1/2 minutes) to run off translating ribosomes before harvest. Soluble extracts were fractionated in the sucrose density gradient by ultracentrifugation. Absorbance at 250 nm was continuously recorded during fraction collection. The relative levels of 80S and polysomes were calculated by quantifying their corresponding peaks in the absorbance profile. Data represent mean +/- SEM (*n* = 3). ***P < .001 to control cells (0 min) as determined by 2-way ANOVA, Tukey’s multiple comparisons test. (**C**) Schematic diagram for puromycin labeling during harringtonine runoff to assess translation rate. (**D, E**) DNAJC2-depleted cells display faster translation elongation than control. siRNA-transfected SH-SY5Y cells were preincubated with the translation initiation inhibitor harringtonine for the indicated time, then treated with puromycin for nascent polypeptide labeling. Puromycin-labeled proteins were quantified by immunoblotting with anti-puromycin antibody (D), and their relative levels were fit to a one-phase decay to calculate a rate constant as a proxy for translation elongation rate (E). Data represent mean +/- SEM (*n* = 3). *P < .05, ***P< .001 as determined by 2-way ANOVA, Tukey’s multiple comparisons test (left) or unpaired t test (right). (**F, G**) DNAJC2 depletion facilitates *ZNF598*-dependent ribosome rescue upon anisomycin-induced ribosome collisions. Control and DNAJC2-depleted cells were incubated with 500 nM anisomycin for the indicated time before harvest (UT, untreated control). The relative levels of 60S, 80S, polysomes, and halfmers were calculated as described in Supplementary Fig. S7B. Data represent mean +/- SEM (*n* = 3). n.s., not significant; *P < .05, **P < .01, ***P < .001 to UT in control (blank) or DNAJC2-depleted cells (pink); to control siRNA at the same time points (purple), as determined by 2-way ANOVA, Tukey’s multiple comparisons test. (**H**) A working model for DNAJC2-dependent control of translation flux and ZNF598-engaged collided ribosome disassembly. HAR and ANS denote harringtonine and anisomycin, respectively, in the figure panels.

We next examined how DNAJC2 depletion affects the behavior of translating ribosomes under collision-prone conditions. Anisomycin treatment of control cells decreased the polysome population while increasing the relative levels of the 60S subunit and 80S monosome over time (Fig. 4F, G; Supplementary Fig. S7A). Halfmer peaks also appeared between the polysome peaks in the light polysome fractions (Fig. 4F, G; Supplementary Fig. S7A, B). Such halfmer peaks indicate mRNAs carrying an additional 40S subunit alongside one or more 80S ribosomes. They can arise when 60S joining is delayed during initiation or when 40S-containing complexes persist after ribosome disassembly (Best et al., 2023, Ford et al., 2025, Lehmann et al., 2024). *ZNF598* deletion suppressed the anisomycin-induced redistribution from polysomes toward 80S and 60S fractions, implicating *ZNF598*-dependent disassembly of collided ribosomes in this process (Fig. 4F, G). Halfmers nevertheless persisted in *ZNF598*-deleted cells. Their presence therefore cannot be used alone as evidence of ZNF598-dependent ribosome rescue and may also reflect stalled or queued 40S-containing initiation complexes in 5’ untranslated regions (UTRs) (Hickey et al., 2020, Juszkiewicz et al., 2020a). DNAJC2-depleted cells consistently exhibited a higher baseline ratio of polysome to 80S monosome. Following anisomycin treatment, these cells showed a greater loss of polysomes, accompanied by enhanced accumulation of 60S subunits and halfmers over time. Strikingly, these effects of DNAJC2 depletion were lost in ZNF598-deleted cells (Fig. 4F, G). This genetic dependence suggests that elevated polysomal content in DNAJC2-depleted cells may increase the likelihood of ribosome collisions and promote ZNF598-dependent RQC. Together, these findings support a model in which DNAJC2 titrates global translational flux to limit excessive polysome loading. This regulation may reduce subsequent ZNF598-dependent ribosome disassembly under collision-prone conditions (Fig. 4H).

### Lowering Translational Flux Rescues the *DNAJC2* Phenotypes on Poly-A Translation

To validate our model above, we designed a set of experiments to rescue the *DNAJC2* phenotypes during RQC-sensitive poly-A translation (Fig. 5A). We limited ribosomal loading onto poly-A translation reporters by (1) low-dose treatment with the reversible translation initiation inhibitor homoharringtonine (Chen et al., 2011b, Fresno et al., 1977, Garreau de Loubresse et al., 2014, Robert et al., 2009, Xing et al., 2023) or (2) the huntingtin upstream open reading frame (uORF) sequence that suppresses downstream ORF translation (Aviner et al., 2024, Lee et al., 2002, Zhang et al., 2019). Homoharringtonine treatment indeed reduced overall reporter expression from the nonstalling K(AAA)_0_ reporter in a dose-dependent manner, but it did not affect the ratio of mCherry-to- GFP expression (Fig. 5B and Supplementary Fig. S8A). In contrast, homoharringtonine treatment relieved translational stalling (ie, mCherry expression relative to GFP) on the RQC-sensitive K(AAA)_20_ reporter, lowering K(AAA)_20_-derived arrested product levels (Fig. 5B, C). The distinct responses of the 2 translation reporters may reflect a lower probability of ribosome collisions in homoharringtonine-treated cells, which likely reduces RQC-relevant ribosome disassembly specifically at the poly-A tract (Hickey et al., 2020, Juszkiewicz et al., 2020a). Furthermore, homoharringtonine treatment suppressed K(AAA)_20_-derived arrested product accumulation more potently in DNAJC2-depleted cells (Fig. 5B, C). To titrate translation flux directly on poly-A translation reporters, we inserted the huntingtin uORF into their 5’ UTRs (Fig. 5D, top). Translation of this short upstream ORF can suppress initiation at the downstream coding sequence, thereby reducing ribosome loading. The huntingtin uORF indeed phenocopied homoharringtonine treatment, dampening translational stalling specifically on the RQC-sensitive K(AAA)_20_ reporter and reducing its arrested product generation, while substantially rescuing the *DNAJC2* phenotypes (Fig. 5D, E; Supplementary Fig. S8B–D). It is unlikely that incomplete ribosome skipping via P2A contributed to the *DNAJC2* phenotypes or the huntingtin uORF effects on poly-A translation reporters since partially skipped reporter proteins were comparably expressed across the genetic conditions tested (Supplementary Fig. S8C, D). The nonadditive interactions between DNAJC2 depletion and translation-flux manipulations in arrested product generation support our model. Specifically, high translation flux in DNAJC2-depleted cells may promote ribosome collisions and enhance the engagement of the ZNF598-initiated RQC pathway. This, in turn, would elevate the burden on co-translational quality control.

**Fig. 5:**
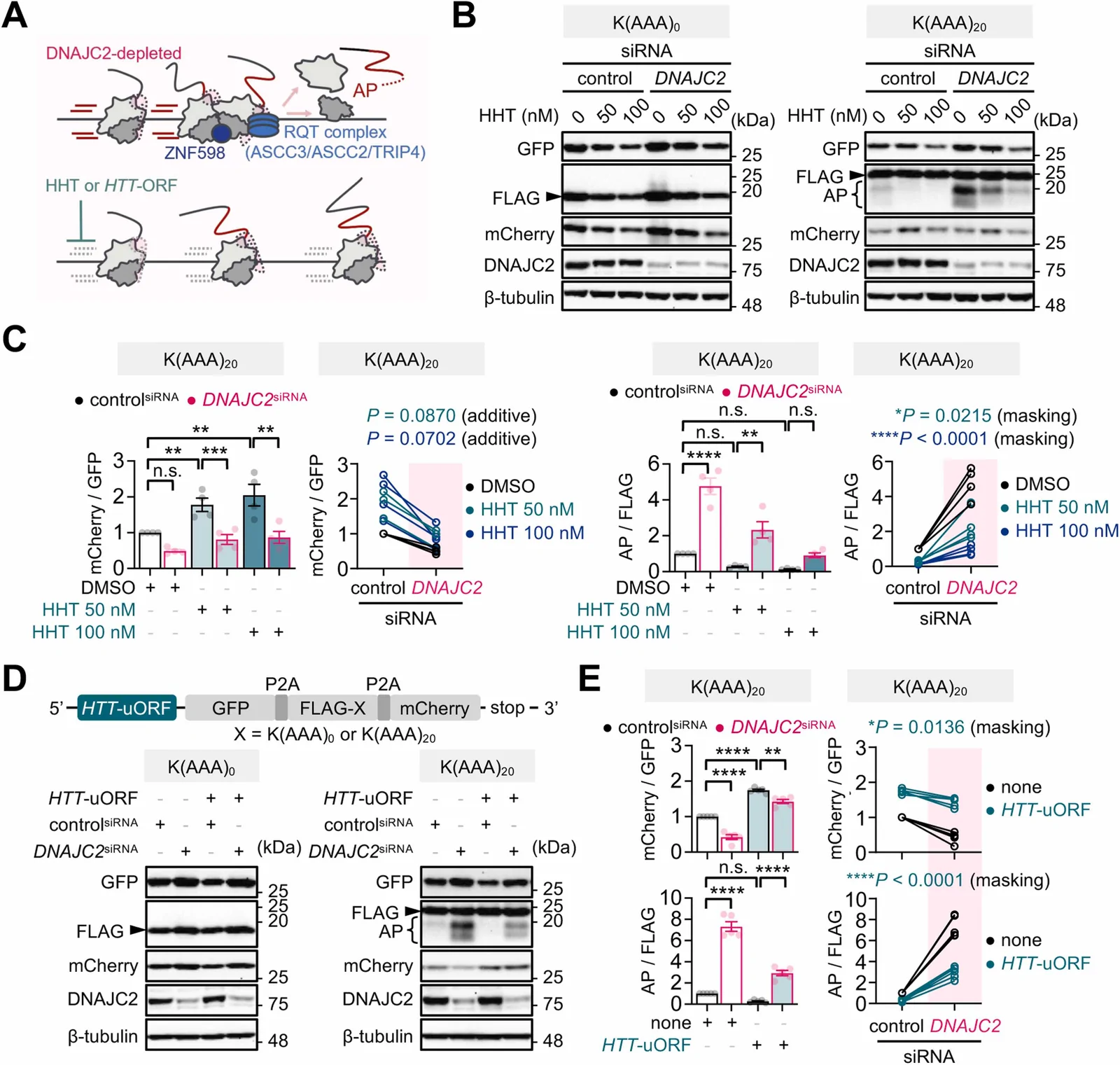
*Lowering translational flux rescues the DNAJC2 phenotypes on poly-A translation.* (**A**) Schematic diagram illustrating the experimental strategies to rescue the *DNAJC2* phenotypes. Homoharringtonine treatment or the huntingtin uORF limits downstream translation flux. (**B, C**) Low-dose homoharringtonine treatment alleviates K(AAA)_20_-derived arrested product accumulation in DNAJC2-depleted cells. Reporter expression was quantified by protein band intensities in immunoblots 12 hours after homoharringtonine or DMSO treatment, similarly as in Figure 1. Two-way ANOVA detected significant interaction effects of DNAJC2 depletion and homoharringtonine treatment on relative arrested product levels to full-length K(AAA)_20_ proteins (AP/FLAG). The interaction *P* values were shown at the top of each graph (green, 50 nM; blue, 100 nM). Data represent mean +/- SEM (*n* = 4); data points connected by lines represent paired measurements from the same experiment. n.s., not significant; **P < .01, ***P < .001, ****P < .0001 as determined by Tukey’s multiple comparisons test. (**D, E**) The huntingtin uORF masks *DNAJC2* effects on poly-A translation. Schematic diagram of the dual-fluorescence reporter containing the 5’ UTR huntingtin uORF sequence. Two-way ANOVA detected significant interaction effects of DNAJC2 depletion and huntingtin uORF on translational stalling (mCherry/GFP) and arrested product generation (AP/FLAG) during the K(AAA)_20_ reporter expression. The interaction *P* values were shown at the top of each graph. Data represent mean +/- SEM (*n* = 5); data points connected by lines represent paired measurements from the same experiment. n.s., not significant; **P < .01, ****P < .0001 as determined by Tukey’s multiple comparisons test. HHT and HTT-uORF denote homoharringtonine and the huntingtin uORF, respectively, in the figure panels.

## DISCUSSION

Co-translational regulation is an elegant cellular strategy for tailoring translation products on ribosomes while triaging aberrant intermediates before ribosomal release. We demonstrate that the ribosome-associated chaperone DNAJC2 limits translation elongation, lowers the probability of ribosome collisions, and thereby titrates the flux of nascent polypeptides toward downstream RQC factors during collision-prone translation. The loss of *DNAJC2* function should saturate RQC capacity, causing excessive proteotoxic burden while sensitizing cells to the *ZAK*-dependent translation stress response. Our work therefore defines *DNAJC2* as an upstream rheostat that modulates collision-dependent engagement of the canonical RQC machinery and RSR signaling, expanding the repertoire of *DNAJC2* function beyond co-translational folding.

Yeast genetic models have been thoroughly explored to elucidate molecular mechanisms underlying RAC function. A broad range of substrates has been documented to associate with the yeast RAC/Ssb complex (ie, >80% of cytoplasmic and nuclear proteins; ∼80% of mitochondrial proteins; >40% of ER-targeted proteins), yet many of these substrates share common molecular features, including large proteins with complex folds (ie, hydrophobic, aggregation-prone, and intrinsically disordered) and residues enriched with positively charged amino acids (Arg or Lys) (Doring and Ahmed, 2017, Willmund et al., 2013). The latter preference may explain RQC-relevant *ZUO1* function, as deletion of the yeast *DNAJC2* homolog *ZUO1* enhances premature termination during poly-A translation and promotes the generation of C-terminally truncated polylysine products (ie, translationally arrested products) in a manner dependent on *HEL2* or *ASC1* (the yeast homologs of *ZNF598* and *RACK1*, respectively) (Chiabudini et al., 2014, Gribling-Burrer et al., 2019). These previous observations thus implicate yeast RAC/Ssb in the RQC pathway, consistent with our findings. Despite genetic and functional connections between the 2 machineries, however, how these ribosome-associated activities cooperatively maintain translational fidelity has remained unclear.

Structural studies have revealed that DNAJC2 homologs interact with both ribosomal subunits (Chen et al., 2022, Kisonaite et al., 2023, Lee et al., 2016, Zhang et al., 2014). The Zuotin homology domain (ZHD) of the yeast DNAJC2 homolog Zuo1 binds to helix H24 of 25S/28S rRNA and ribosomal protein eL31 near the peptide tunnel exit at the 60S subunit. This interaction positions Zuo1 to support co-translational folding by delivering nascent chains to the Hsp70/Ssb chaperone. On the other hand, the C-terminal 4-helix bundle domain of DNAJC2 interacts with the expansion segment 12 (ES12) of 18S rRNA at the 40S subunit. Given that ES12 corresponds to the peripheral end of the helix H44 at the decoding center, it has been thought that RAC influences translation fidelity and possibly prevents premature termination through the RAC-40S interface (Chen et al., 2022, Gribling-Burrer et al., 2019, Kisonaite et al., 2023). Because DNAJC2 spans the 2 ribosomal subunits, loss of this intersubunit constraint may facilitate ribosome rotation and thereby accelerate translation elongation in DNAJC2-depleted cells (Kisonaite et al., 2023, Lee et al., 2016, Zhang et al., 2014). Prior yeast genetic studies have provided indirect evidence suggesting that loss of RAC/Ssb function may reduce, rather than increase, translation flux (Doring and Ahmed, 2017, Rodriguez-Galan et al., 2021). Altered elongation dynamics have been proposed to contribute to premature termination during collision-prone translation in RAC/Ssb-deficient yeast models (Chiabudini et al., 2014). However, direct experimental evidence linking ribosomal flux regulation to co-translational quality control has remained limited. In this regard, our study provides important mechanistic support for the rheostat model that faster translation in DNAJC2-depleted cells enhances premature translation termination at ZNF598-sensitive stalling sequences.

We speculate that RQC-relevant RAC function may have evolved in quite distinct translational contexts between yeast and humans. Zuo1 requires 2 distinct Hsp70 partners for chaperone function (ie, Ssz1 and Ssb), whereas mammalian DNAJC2 associates with the Ssz1 homolog HSPA14 to assemble a functional RAC (Deuerling et al., 2019, Gamerdinger and Deuerling, 2024, Hundley et al., 2005, Kramer et al., 2019, Otto et al., 2005). Mammalian *DNAJC2* exclusively encodes a C-terminal SANT domain for nonribosomal functions such as chromatin targeting and epigenetic gene regulation. We should further add that the relative abundance of Zuo1 in yeast is 10-fold higher than that of human DNAJC2 (https://pax-db.org/) (Huang et al., 2023), likely correlating with the functional divergence of RAC homologs among different species (Gamerdinger, 2016). For instance, RAC/Ssb deletion promotes ribosomal particle aggregation and lowers relative levels of the 80S and polysomal populations, reducing translation activity (Chiabudini et al., 2014, Koplin et al., 2010). Ribosomal halfmers are readily seen in RAC-/Ssb-deficient ribosomal populations, indicating defective 60S assembly and the accumulation of 48S preinitiation complexes due to an imbalance between 40S and 60S subunits (Albanese et al., 2010, Koplin et al., 2010). In stark contrast, we found that human DNAJC2 depletion does not generate halfmers under baseline conditions but rather elevates polysome levels with fast translation elongation (Wu et al., 2021). This phenotypic difference supports the possibility that mammalian *DNAJC*2 has lost its direct roles in ribosomal biogenesis but acquired other activities during evolution.

Taken together, our findings identify mammalian DNAJC2 as a ribosome-associated regulator that couples translational flux control to ribosome collision surveillance. By restraining elongation under collision-prone conditions, DNAJC2 may buffer ribosome traffic and thereby preserve productive protein synthesis while limiting excessive activation of RQC and translation stress signaling. This function extends RAC beyond its canonical role in co-translational folding, suggesting that RAC may help set the threshold at which altered elongation dynamics are interpreted as quality control cues. Although our analyses of XBP1u-derived reporters suggest that DNAJC2 regulates arrested product accumulation at a physiologically relevant stalling sequence, whether DNAJC2 contributes to the quality control of endogenous RQC substrates and affects the associated physiological processes remains to be established. Future studies should define specific molecular substrates and distinct physiological contexts in which RQC-relevant DNAJC2 function supports proteostasis and cellular stress adaptation.

## CRediT authorship contribution statement

**Jongmin Park:** Writing---original draft, visualization, validation, methodology, investigation, formal analysis, and conceptualization. **Youngim Yu:** Visualization, methodology, investigation, and formal analysis. **Heeju Park:** Validation, methodology, investigation, and formal analysis. **Hyunwoo Bang:** Validation, methodology, investigation, and formal analysis. **Jongbin Lee:** Methodology. **Chunghun Lim:** Writing---review and editing, supervision, funding acquisition, and conceptualization.

## Data Availability

All data are presented in the main text or in the supplementary data.

## Declaration of Generative AI and AI-Assisted Technologies in the Writing Process

During the preparation of this work, the authors used ChatGPT to organize information from the primary literature and improve the language and clarity of the paper. All AI-assisted content was reviewed and edited by the authors, who take full responsibility for the paper.

## Declaration of Competing Interest

The authors declare that they have no known competing financial interests or personal relationships that could have appeared to influence the work reported in this paper.

## Footnotes

Appendix A Supplementary data associated with this article can be found in the online version at doi:10.1016/j.mocell.2026.100407.
