# Supplementary material for: Ribosome-associated chaperone DNAJC2 titrates translation flux to alleviate the burden of co-translational quality control
Source: Mol Cells. 2026 Oct 14;49(12):100407. doi: 10.1016/j.mocell.2026.100407 (PMC13757519; doi:10.1016/j.mocell.2026.100407)
Supplement: Supplementary file 1 [file mmc1.pdf]

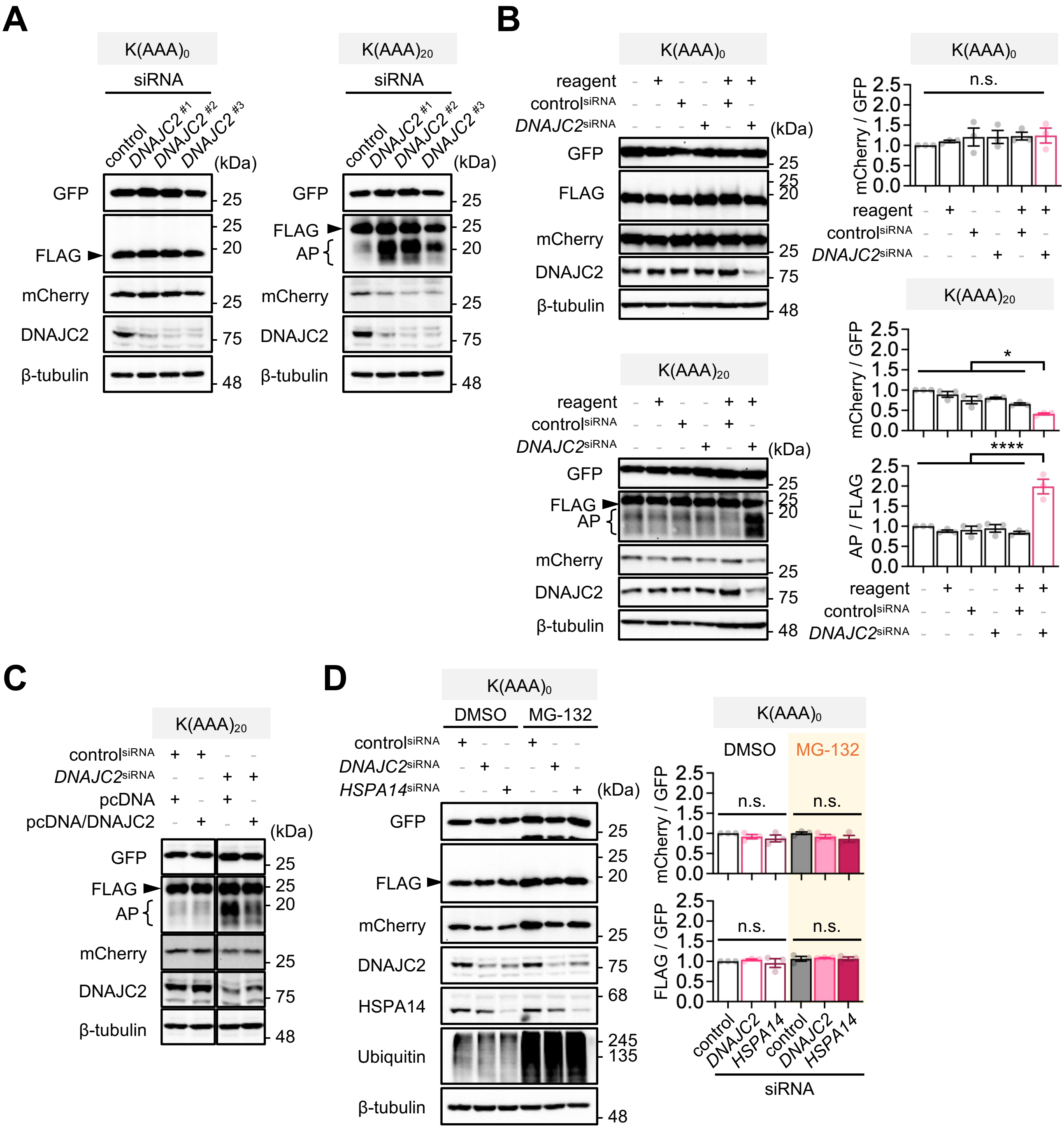

Supplementary Figure 1. DNAJC2 depletion enhances translational stalling on the K(AAA)$_{20}$ reporter and AP accumulation. (**A**) Independent *DNAJC2* siRNAs consistently cause translational stalling on the K(AAA)$_{20}$ reporter while elevating AP levels. (**B**) Transfection reagent per se does not affect poly-A translation. Reporter protein expression was assessed by immunoblotting, similarly as in Fig. 1. Data represent mean +/- SEM (n = 3). n.s., not significant; *$P < 0.05$, ****$P < 0.0001$ as determined by 1-way ANOVA, Tukey's multiple comparisons test. (**C**) DNAJC2 overexpression reduces K(AAA)$_{20}$-derived AP levels in DNAJC2-depleted cells. siRNA-transfected cells were further co-transfected with expression vectors for K(AAA)$_{20}$ reporter and wild-type DNAJC2 cDNA (pcDNA/DNAJC2) 48 h before harvest. (**D**) RAC depletion does not affect the K(AAA)$_0$ reporter expression regardless of MG-132 treatment. Data represent mean +/- SEM (n = 3). n.s., not significant; as determined by 2-way ANOVA, Tukey's multiple comparisons test.

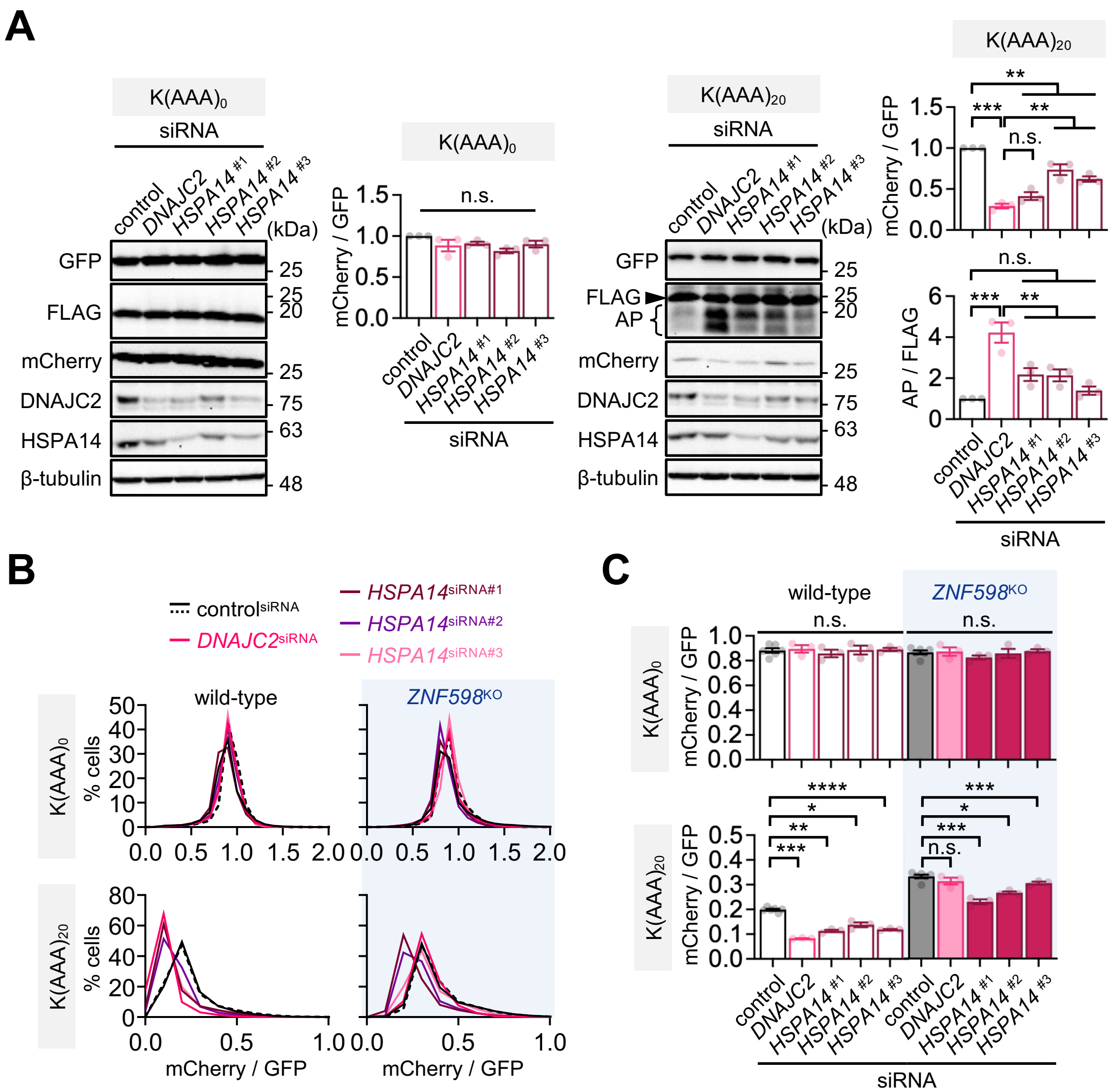

Supplementary Figure 2. HSPA14 depletion affects $K(AAA)_{20}$ reporter expression in a manner distinct from DNAJC2 depletion. (**A**) HSPA14 depletion causes modest phenotypes in translational stalling and AP generation during $K(AAA)_{20}$ expression, compared to DNAJC2 depletion. Reporter protein expression was assessed by immunoblotting, similarly as in Fig. 1. Data represent mean +/- SEM (n = 3). n.s., not significant; $^{**}P < 0.01$, $^{***}P < 0.001$, as determined by 1-way ANOVA, Tukey's multiple comparisons test. (**B, C**) *HSPA14* depletion enhances translational stalling on the K(AAA)20 reporter in both control and *ZNF598*-deleted cells. Histograms show the percentages of cells with the given mCherry-to-GFP expression ratio for each translation reporter. The median ratio values were determined from independent experiments [n = 6 for $\text{control}^{\text{siRNA}}$; n = 3 for other siRNAs] and compared across different genetic backgrounds. Two-way ANOVA detected significant interaction effects of $\text{ZNF598}^{\text{KO}}$ with $\textit{DNACJ2}^{\text{siRNA}}$ (P = 0.0014) and $\textit{HSPA14}^{\text{siRNA\#3}}$ (P = 0.0002) on the $K(AAA)_{20}$ reporter expression. Data represent mean +/- SEM. n.s., not significant; $^{*}P < 0.05$, $^{**}P < 0.01$, $^{***}P < 0.001$, $^{****}P < 0.0001$ as determined by Tukey's multiple comparisons test.

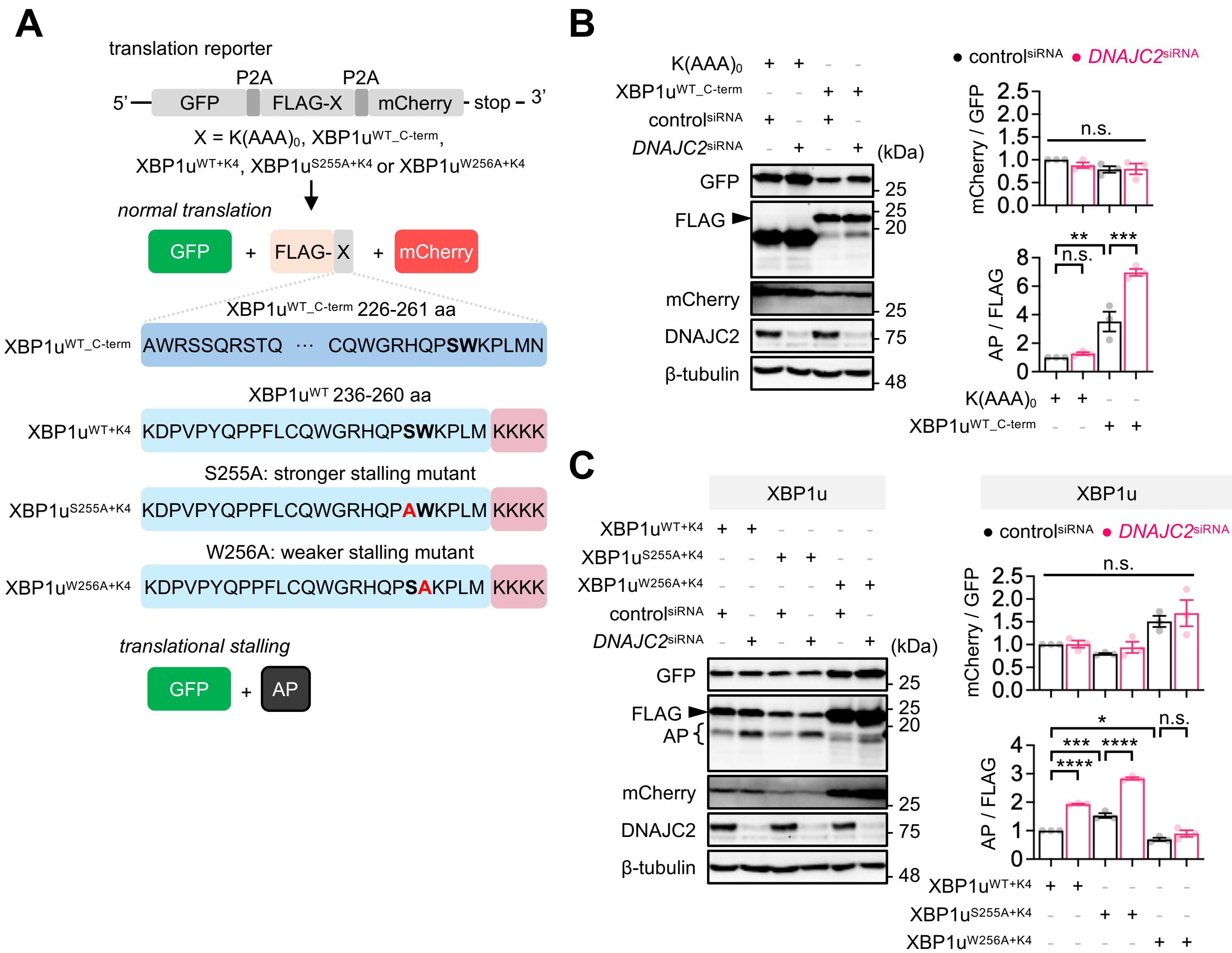

Supplementary Figure 3. DNAJC2 depletion enhances AP accumulation from XBP1u stalling reporters. **(A)** Schematic of the dual-fluorescence reporter used to assess translational stalling and AP generation during XBP1u translation. The human XBP1u wild-type C-terminal sequence (WT_C-term; residues 226–261) or the wild-type, S255A, or W256A stalling sequence (residues 236–260), each followed by four lysine residues (+K4), was inserted at the position marked X. **(B)** DNAJC2 depletion enhances AP accumulation from the reporter containing the wild-type XBP1u C-terminal sequence compared to the K(AAA)$_0$ control. **(C)** WT+K4 or S255A+K4 XBP1u reporters show enhanced AP accumulation upon DNAJC2 depletion, whereas the W256A+K4 reporter shows no significant change. Reporter protein expression was assessed by immunoblotting as in Fig. 1. Data represent mean +/- SEM (n = 3). n.s., not significant; *P < 0.05, **P < 0.01, ***P < 0.001, ****P < 0.0001 as determined by 2-way ANOVA, Tukey's multiple comparisons test.

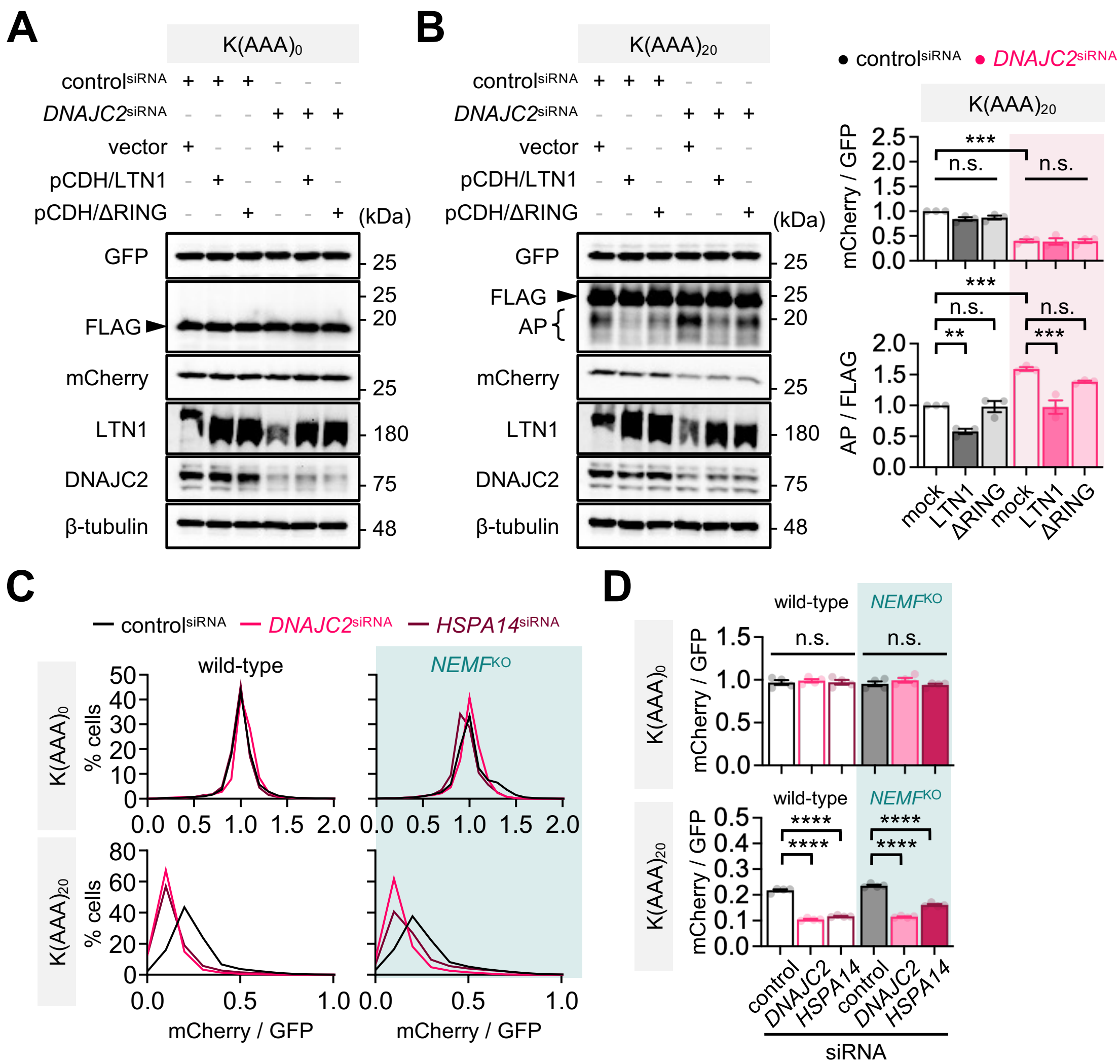

Supplementary Figure 4. RAC-sensitive translational stalling on K(AAA)$_{20}$ reporter is unlikely to involve *LTN1* or *NEMF*. (**A, B**) LTN1 overexpression does not affect *DNAJC2*-sensitive translational stalling during K(AAA)$_{20}$ expression while reducing K(AAA)$_{20}$-derived AP levels in both control and DNAJC2-depleted cells. Reporter protein expression in different genetic backgrounds was assessed by immunoblotting similarly as in Fig. 1. Two-way ANOVA detected no significant interaction effects between DNAJC2 depletion and LTN1 overexpression on the K(AAA)$_{20}$ reporter expression. Data represent mean +/- SEM (*n* = 3). n.s., not significant; $^{**}P < 0.01$, $^{***}P < 0.001$ as determined by Tukey's multiple comparisons test. ΔRING, a LTN1-deletion mutant defective in E3 ligase activity. (**C, D**) *NEMF* deletion does not mask RAC-depletion phenotype in translational stalling on the K(AAA)$_{20}$ reporter. Histograms show the percentages of cells with the given mCherry-to-GFP expression ratio for each translation reporter. The median ratio values were determined from independent experiments and compared across different genetic backgrounds. Two-way ANOVA detected no significant interaction effects between DNAJC2 depletion and *NEMF*^{KO} on the K(AAA)$_{20}$ reporter expression. Data represent mean +/- SEM (*n* = 4). n.s., not significant; $^{****}P < 0.0001$ as determined by Tukey's multiple comparisons test.

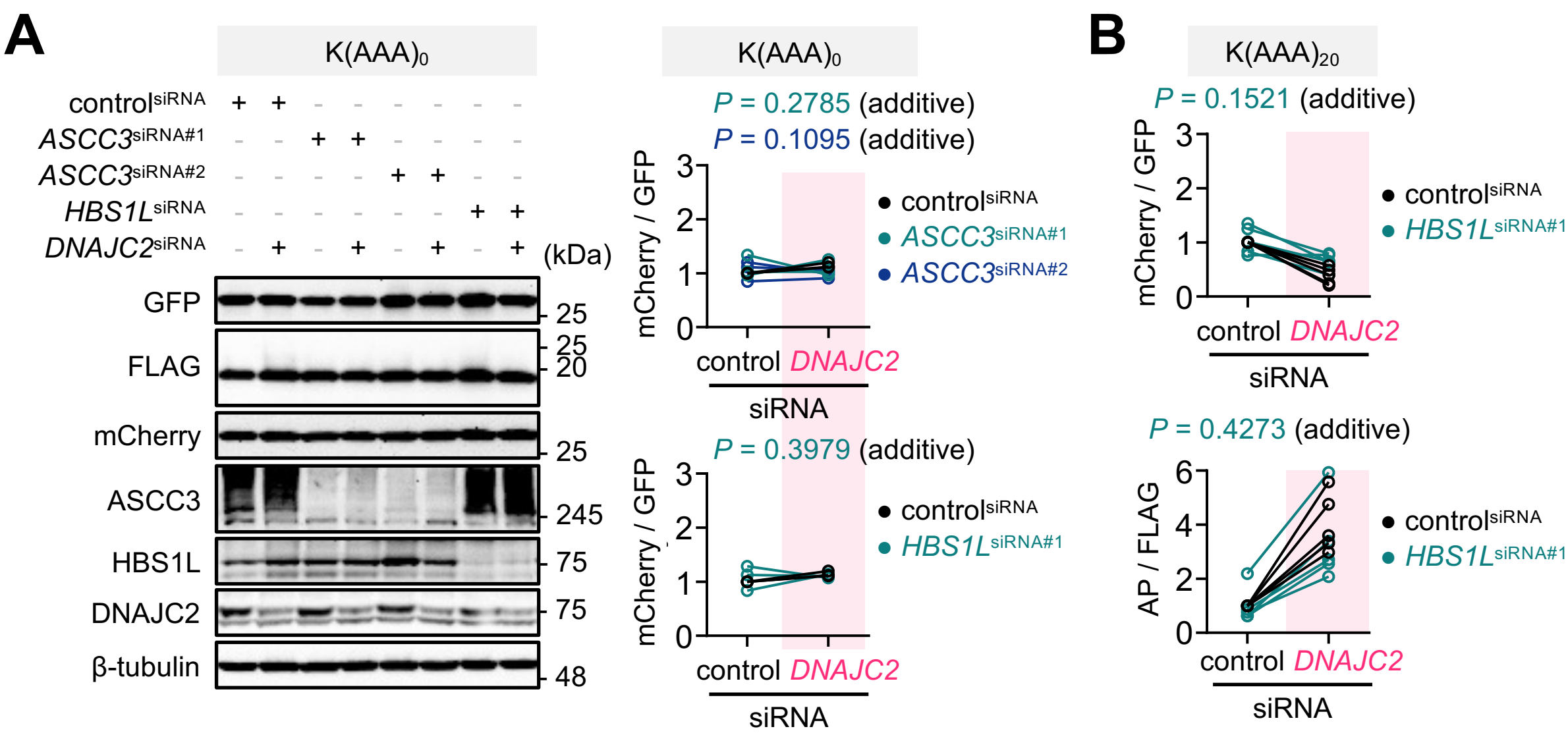

Supplementary Figure 5. ASCC3 deficiency reduced AP generation in DNAJC2-depleted cells and attenuated translational stalling on the K(AAA)20 reporter. (**A**) Depletion of DNAJC2 and ribosome rescue factors (ASCC3, HBS1L) has no effect on the $K(AAA)_0$ control reporter expression. The interaction *P* values from the 2-way ANOVA were shown at the top of each graph. Data points connected by lines represent paired measurements from the same experiment (*n* = 3). (**B**) HBS1L depletion does not affect *DNAJC2* effects on the $K(AAA)_{20}$ reporter expression. The interaction *P* values from the 2-way ANOVA were shown at the top of each graph. Data points connected by lines represent paired measurements from the same experiment (*n* = 5).

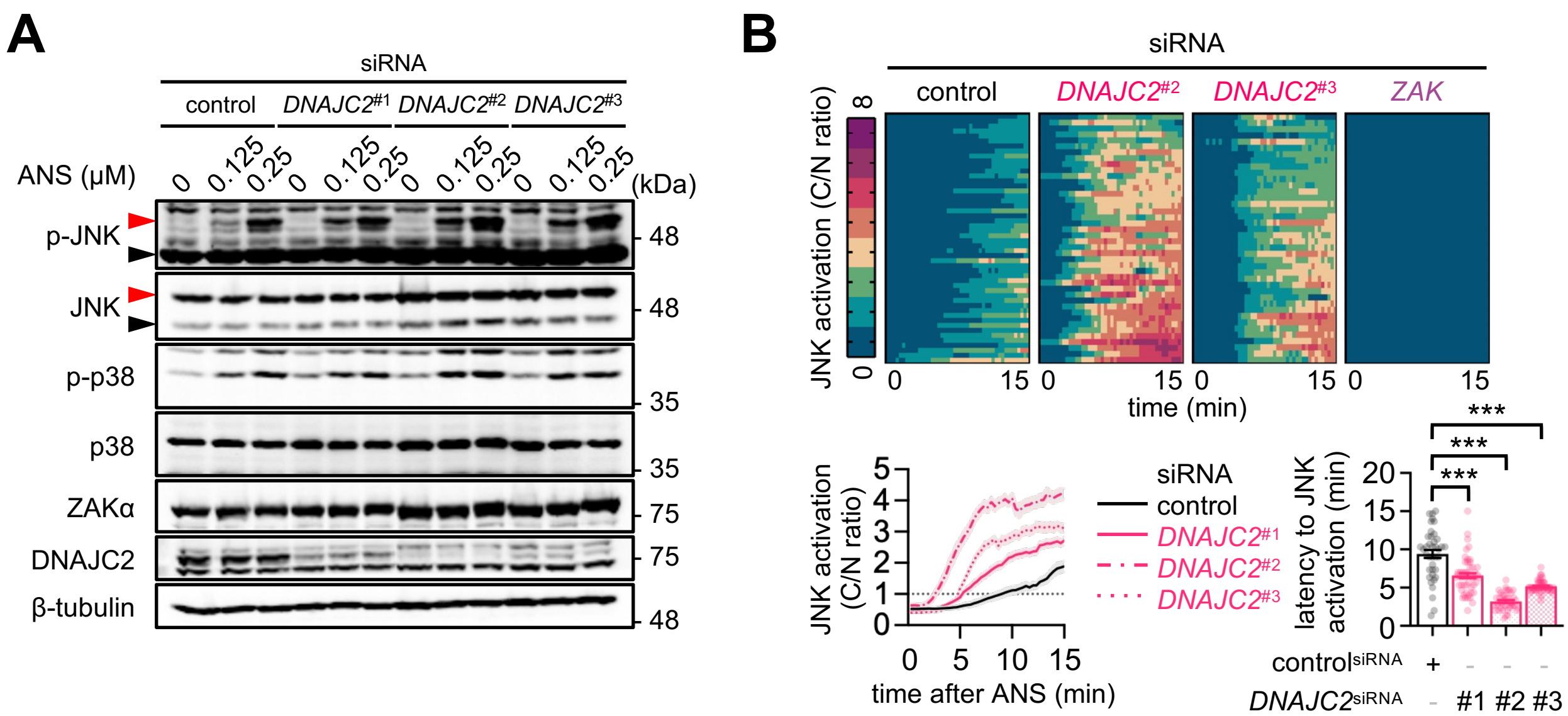

Supplementary Figure 6. DNAJC2 limits translation flux and ribosome collision-induced RSR activation. (**A**) Independent *DNAJC2* siRNAs consistently facilitate RSR activation upon ANS-induced ribosome collision. Immunoblotting of total lysates from ANS-treated SH-SY5Y cells was performed as in Fig. 3A. Red arrowheads, p54 isoform; black arrowheads, p46 isoform. (**B**) Independent *DNAJC2* siRNAs consistently promote ANS-induced JNK-KTR activation. The C/N ratio of the fluorescence intensity was calculated in individual cells over time and displayed as a heatmap (top) or as the average (bottom). Latency to JNK activation was determined in individual cells as described in Fig. 3C. *ZAK* siRNA served as a control to validate *ZAK*-dependent JNK-KTR activation. Data represent mean +/- SEM (*n* = 39–51). ***$P < 0.001$ as determined by ART 1-way ANOVA, Wilcoxon rank sum test.

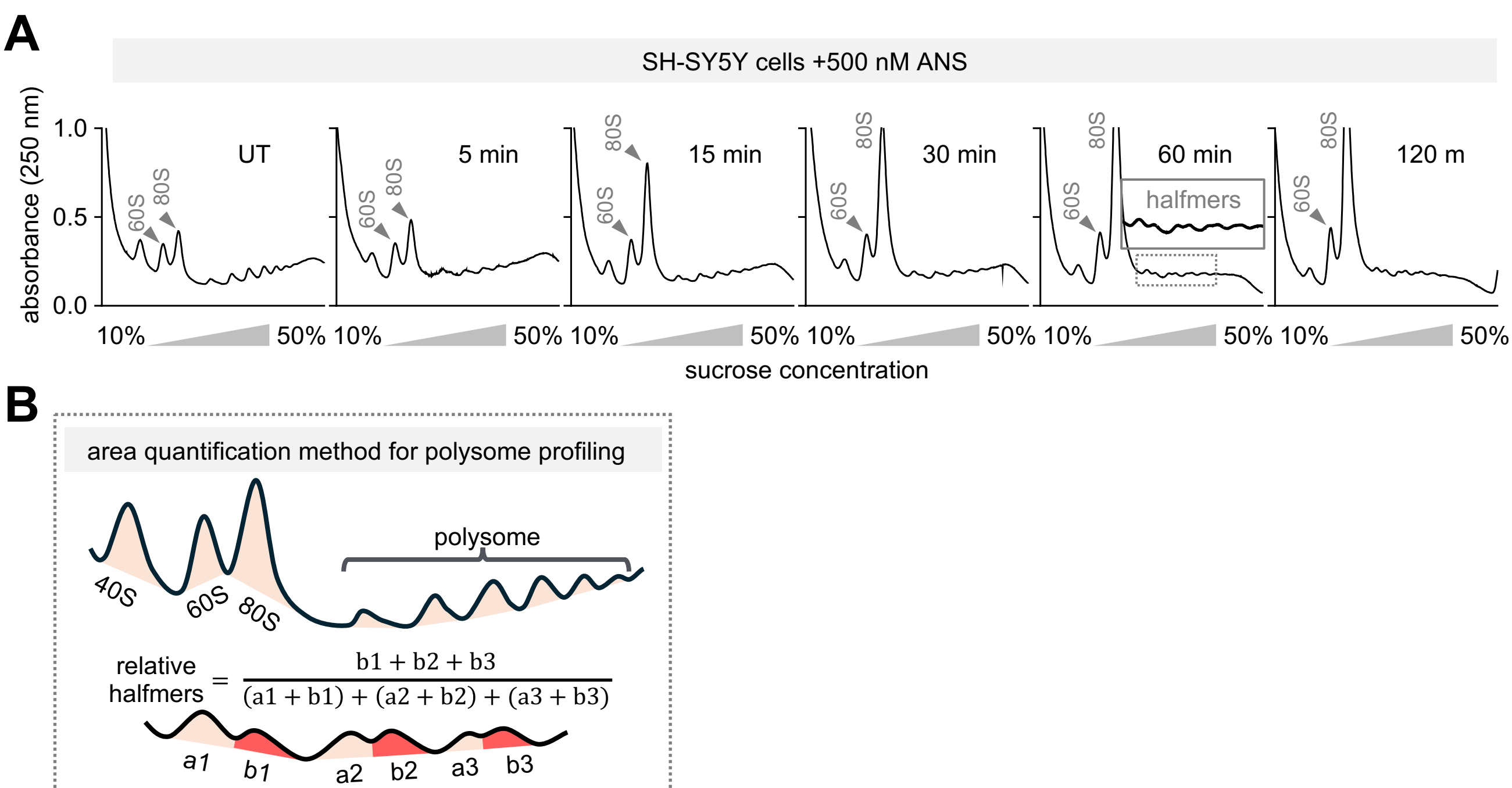

Supplementary Figure 7. Anisomycin treatment dissociates polysomes over time while accumulating 60S subunit, 80S monosome, and light-polysomal halfmers. (**A**) Wild-type SH-SY5Y cells were incubated with 500 nM ANS for the indicated time before biochemically fractionating their lysates in the sucrose density gradient. UT, untreated control cells. (**B**) Schematic of area-based quantification for polysome profiles. Relative halfmer levels were calculated by normalizing the sum of halfmer peak areas (red) to the total polysomal area, including halfmer peaks (cream + red).

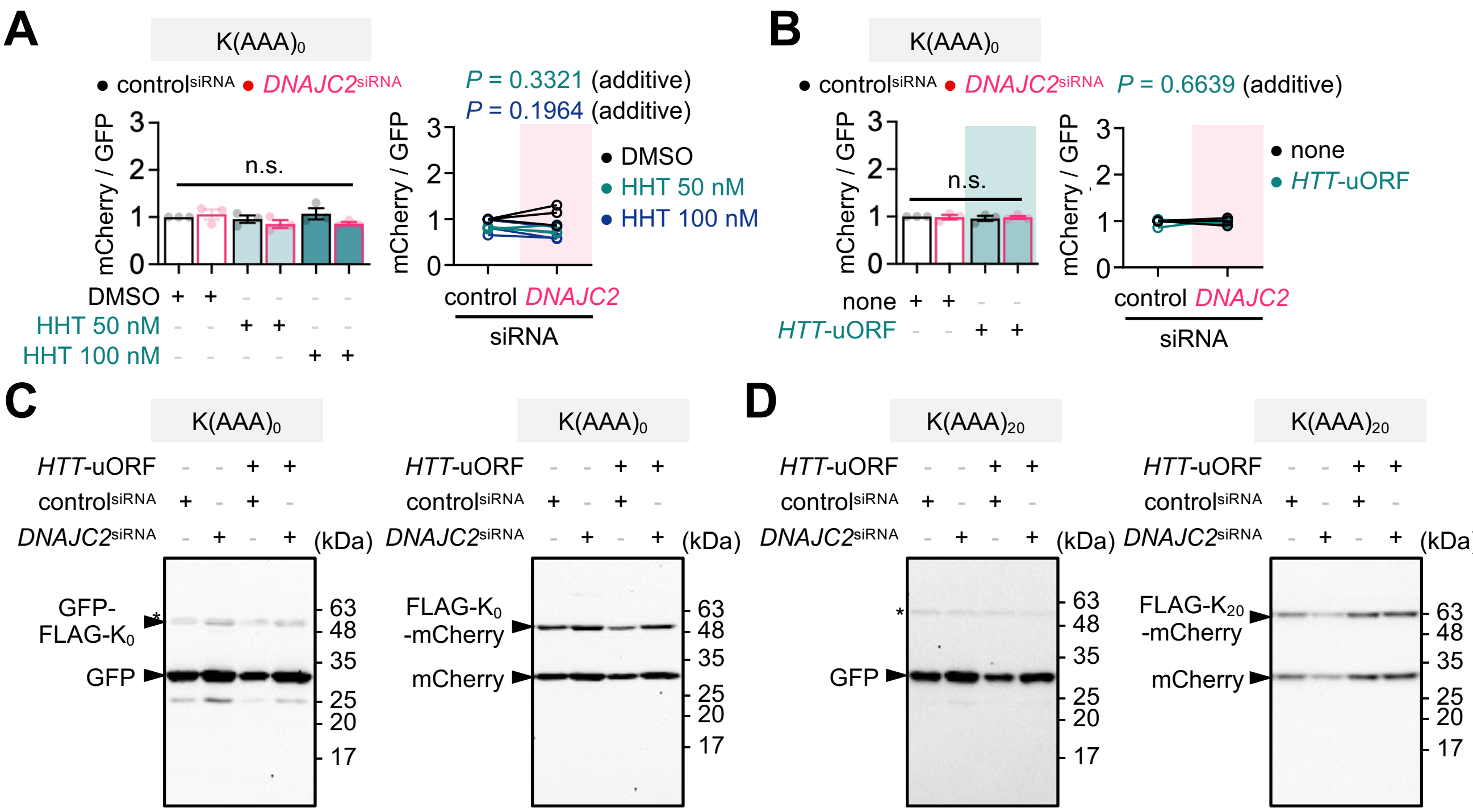

Supplementary Figure 8. Limiting translation flux or ribosome disassembly rescues the *DNAJC2* phenotypes on poly-A translation. (**A**) HHT treatment does not affect the ratiometric expression of fluorescence reporter proteins (mCherry/GFP) from the non-stalling $K(AAA)_0$ reporter. Data represent mean +/- SEM (n = 3); data points connected by lines represent paired measurements from the same experiment. The interaction P values from the 2-way ANOVA were shown at the top (green, 50 nM HHT; blue, 100 nM HHT). n.s., not significant as determined by Tukey's multiple comparisons test. (**B–D**) The *HTT* upstream ORF (*HTT*-uORF) affects neither the ratiometric expression of fluorescence reporter proteins (mCherry/GFP) from the non-stalling $K(AAA)_0$ reporter nor ribosome-skipping P2A efficiency across experimental conditions tested. Uncropped full-blot images of Fig. 5D were shown. Arrowheads, target-specific; asterisks, non-specific. Data represent mean +/- SEM (n = 3); data points connected by lines represent paired measurements from the same experiment. The interaction P value from the 2-way ANOVA was shown at the top. n.s., not significant as determined by Tukey's multiple comparisons test.
